# Exploring psychosocial barriers in digital walking interventions for the chronically ill: a scoping review

**DOI:** 10.3389/fpsyg.2026.1869579

**Published:** 2026-07-17

**Authors:** Regina Subach, Greta Zanchi, Valentina Carfora, Patrizia Catellani

**Affiliations:** Department of Psychology, Catholic University of the Sacred Heart, Milan, Italy

**Keywords:** chronic illness, digital health interventions, patient engagement, psychosocial barriers, walking

## Abstract

**Objective:**

This scoping review aimed to identify and systematically map existing evidence on psychosocial barriers in digital physical activity interventions promoting walking among individuals with chronic illness.

**Introduction:**

Digital interventions promoting walking are widely used in chronic disease management and demonstrate short-term effectiveness. Psychosocial barriers, such as stigma, illness-related beliefs, and internalized meanings, may play a critical role in shaping both intervention engagement and behavioral outcomes, yet their conceptualization and integration into intervention design remain unclear.

**Inclusion criteria:**

Quantitative and qualitative studies on psychosocial barriers in digital physical activity interventions focusing on walking among individuals with chronic illness.

**Methods:**

A three-step search strategy was conducted across PubMed, Web of Science, Scopus, and PsycINFO, complemented by gray literature. Studies in English with no date restrictions were included. Screening was conducted according to predefined inclusion criteria, with backward citation searching applied. Data on study characteristics, participants, intervention features, and psychosocial barriers were extracted. Descriptive statistics and qualitative synthesis were used to map barrier domains.

**Results:**

A total of 28 studies were included, resulting in 105 psychosocial barrier instances grouped into seven domains. The most frequent were belief-based (21.0%) and emotional barriers (21.0%), followed by self-regulation-related barriers (15.2%). Barriers were predominantly identified in qualitative and mixed-methods studies, indicating an exploratory evidence base. Outcome mapping showed that barriers were commonly reported in relation to intervention-process outcomes (54.3%), including engagement, adherence, and usability, followed by behavioral outcomes (28.6%) such as walking and physical activity, and psychological and self-management outcomes (14.3%). Belief-based, digital capability, and emotional barriers were primarily associated with intervention-process outcomes such as engagement, adherence, and acceptability. In contrast, walking-related outcomes were most frequently mapped alongside belief-based, motivational, emotional, and relational factors.

**Conclusions:**

Psychosocial barriers in digital walking interventions operate as multi-level, functionally distinct mechanisms influencing both engagement and behavior. Current evidence is conceptually rich but methodologically heterogeneous and predominantly process-oriented. Future interventions should move beyond generic behavior change techniques to explicitly target psychosocial barriers to improve sustained engagement.

**Systematic review registration:**

https://osf.io/2hrmy.

## 1 Introduction

Digital Physical Activity (PA) interventions are increasingly implemented among individuals with chronic illnesses (Kirk et al., 2019; Kwan et al., 2020) and have demonstrated effectiveness in improving PA indicators and clinical markers, including cardiovascular fitness and metabolic parameters (Bravata et al., 2007; Heizmann et al., 2023; Kirk et al., 2019). Wearable devices that provide feedback have been associated with increases in short-term daily walking activity and improvements in aerobic or physical capacities among cardiovascular patients. These devices may also support secondary prevention when integrated with cardiac rehabilitation services (Ashur et al., 2021; Heizmann et al., 2023). Similarly, pedometers and accelerometers, when used as motivational tools in PA programs, can modestly improve glycaemic markers and steps in patients with type 2 diabetes (de Oliveira et al., 2024). Among patients with chronic obstructive pulmonary disease (COPD), wearable monitors used alongside behavior change techniques, such as goal setting and self-monitoring, and have also been suggested to improve step-driven activity (Reilly et al., 2023).

Although clinical trials report initial efficacy and short-term improvements in PA outcomes (de Oliveira et al., 2024; Heizmann et al., 2023), sustaining engagement remains a challenge. This limitation may be partly explained by the tendency of digital interventions to focus primarily on behavioral techniques such as goal setting, automated reminders, feedback loops, and self-monitoring (Karekla et al., 2019; Liu et al., 2024; McQuaid and Doyle, 2018). However, less attention has been given to psychosocial and contextual factors that may shape sustained use, including interpersonal, sociocultural, environmental, and illness-related barriers (Karekla et al., 2019). This has limited the extent to which digital interventions are designed and tailored around user factors that shape engagement and sustained use. Integrating standardized, framework-based designs with more personalized strategies that address psychological friction (i.e., mental resistance) is relevant for activating engagement among chronically ill patients (Park et al., 2024; Sanabria et al., 2023; Weber et al., 2021). So far, the most studied psychosocial barriers that make sustained use or walking behavior more difficult are motivational decline, technology acceptance issues, and fear-based psychological barriers (Abaraogu et al., 2018; Bartlett et al., 2017; Bradbury et al., 2019; Moy et al., 2016; Richardson et al., 2010; Robinson et al., 2023; Wulfovich et al., 2019). Important gaps remain in understanding stigma, illness-related beliefs, and internalized meanings within intervention contexts (Montesano et al., 2025; Moy et al., 2016).

Psychosocial barriers are also addressed inconsistently in the literature (Greenhalgh et al., 2017; Murray et al., 2016). Some studies explicitly conceptualize them as barriers or challenges, while others report them indirectly as predictors of disengagement, moderators of intervention effects, or explanatory factors identified in process evaluations (Craig et al., 2008; Sekhon et al., 2017). This reflects a broader theoretical gap in the literature, resulting from the absence of a shared framework for defining and classifying psychosocial barriers across studies.

Furthermore, evidence on psychosocial barriers is spread across multiple research designs and varies substantially in terminology, theoretical framing, and measurement approaches. For instance, randomized controlled trials often report secondary findings on engagement barriers (Brosseau et al., 2012; McCartney et al., 2025; Moy et al., 2016; Richardson et al., 2010; Sheshadri et al., 2020), whereas qualitative studies explore patient experiences within digital PA intervention contexts (Dennison et al., 2013; Park et al., 2024) and mixed-methods process evaluations address digital self-management in chronic illness populations (Webb et al., 2019).

Taken together, these inconsistencies limit clarity about how psychosocial barriers are conceptualized, operationalized, and integrated into the design of digital walking interventions.

Several recent reviews have examined topics related to psychosocial barriers in digital PA interventions for individuals with chronic conditions. For example, Kwan et al. (2020) reviewed barriers and facilitators to health technology adoption among older adults with chronic diseases, including telemedicine, wearables, and mobile health. However, their review addressed digital health technologies broadly and was not limited to walking-based interventions. Similarly, Kim et al. (2021) examined determinants of adherence to self-guided digital interventions among adults with cancer but focused on digital programs in general rather than walking-specific interventions. Abe et al. (2026) explored older adults' perceptions of web- and mobile-based PA interventions, addressing user experience and participation issues relevant to psychosocial factors. However, their review did not focus on populations with diagnosed chronic conditions. Kirk et al. (2019) analyzed how engagement is defined and measured in mHealth interventions for people with chronic health conditions but concentrated on engagement metrics rather than psychosocial barriers.

In summary, although some reviews have discussed various aspects of digital health (Abe et al., 2026; Albergoni et al., 2019; Kim et al., 2021; Kirk et al., 2019; Kwan et al., 2020; Valenzuela et al., 2018), none has specifically examined psychosocial barriers within digital walking interventions for individuals with chronic illness as the primary focus. Addressing this gap is particularly relevant because walking is one of the most accessible and scalable forms of PA for individuals with chronic conditions (Lee and Buchner, 2008). Digital walking interventions are widely used in self-management and rehabilitation contexts (Stavric et al., 2022), yet, their design may need to account more explicitly for psychosocial constraints that affect sustained use (Murray et al., 2016).

Accordingly, this review aimed to map psychosocial barriers within digital walking interventions to clarify their conceptualization and inform the design of more responsive and effective interventions. The review included both adults (≥18) and older adults (≥65) with chronic illnesses to support conceptual coherence and capture psychosocial barriers across populations commonly targeted by chronic care services. This decision also enhanced conceptual breadth, as older adults may face distinct psychosocial and technology-related barriers. Furthermore, to reinforce conceptual clarity and precision in mapping psychosocial barriers while reducing behavioral heterogeneity, the review was restricted to digital walking interventions in which walking was a primary or central PA target.

## 2 Review questions

*Primary:*

1. What psychosocial barriers are identified among chronically ill individuals in digital walking interventions?

*Secondary:*

2. How are psychosocial barriers conceptually framed (e.g., as individual, interpersonal, or contextual factors) within digital walking interventions?
3. How are psychosocial barriers operationalized across studies?
4. What outcomes or process indicators are reported in relation to psychosocial barriers in digital walking interventions?

## 3 Inclusion criteria

The eligibility criteria were defined as a priori using the Population Concept Context (PCC) framework (Peters et al., 2020).

### 3.1 Participants

Adults (≥18 years) and older adults (≥65 years) with one or more chronic illnesses (e.g., cardiovascular diseases, diabetes, chronic respiratory diseases, musculoskeletal conditions, cancer survivorship, neurological, or metabolic conditions). Exclusion criteria included studies focusing exclusively on children or adolescents, or populations without a chronic illness diagnosis, or samples composed solely of healthcare professionals or caregivers.

### 3.2 Concept

Psychosocial barriers within digital PA interventions aimed at promoting walking. This includes, but is not limited to, barriers related to motivation, self-efficacy, identity, age- or illness-related stereotypes, stigma, perceived norms, beliefs, and attitudes influencing walking behavior and engagement with digital tools. Psychosocial barriers within digital walking interventions were defined as psychological, social, relational, contextual, or digitally mediated factors that limited, discouraged, complicated, or reduced engagement with walking behavior or with the digital intervention supporting walking. This included, but was not limited to, barriers related to motivation, self-efficacy, identity, illness- or age-related stereotypes, stigma, perceived norms, beliefs, emotions, attitudes, social support, perceived resources, and digital confidence. Digital capability and usability barriers were included only when the technology-related issue had a psychosocial component, such as low digital literacy, low confidence using the device, perceived burden, frustration, distrust in feedback, reduced acceptability, or disengagement. Studies addressing only physical, environmental, or purely technological barriers, such as device malfunction, connectivity problems, or infrastructure limitations, without a psychosocial component were excluded, as were interventions promoting PA broadly without reference to walking. Exclusion criteria included studies addressing only physical, environmental, or technological barriers (e.g., infrastructure, device usability) without a psychosocial component, or interventions promoting PA broadly without reference to walking.

### 3.3 Context

Digital walking interventions (e.g., mobile apps, wearables, web-based platforms, and SMS-based programs) delivered in community, home-based, outpatient, or primary care settings, across all geographic locations and cultural contexts. No restrictions were applied regarding country, healthcare system, gender, or cultural background. Exclusion criteria included non-digital or purely face-to-face interventions or multi-component interventions where walking was not the primary objective or central component; laboratory-based studies with no real-world or applied context; or interventions implemented exclusively in inpatient or institutionalized settings (e.g., long-term residential care) unless digital and walking-focused.

## 4 Type of evidence sources

This scoping review includes a broad range of study designs to comprehensively map the available evidence. Eligible sources were experimental and quasi-experimental studies (such as randomized controlled trials, non-randomized controlled trials, before-and-after studies, and interrupted time-series studies), as well as analytical observational studies (including prospective and retrospective cohort studies, case-control studies, and analytical cross-sectional studies). Descriptive observational designs (such as case series, individual case reports, and descriptive cross-sectional studies) and qualitative studies were also eligible. Reviews, such as systematic reviews and meta-analyses that meet the inclusion criteria, were considered only for supplementary searching by screening their reference lists to identify additional relevant primary studies. Only studies published in English were included. Publications in other that English languages were excluded. No publication year restrictions were applied.

## 5 Methods

This scoping review was conducted in accordance with the JBI methodology for scoping reviews (Peters et al., 2020) and reported following the PRISMA-ScR guidelines (Tricco et al., 2018) to ensure methodological transparency and completeness.

### 5.1 Search strategy

The search strategy aimed to identify both published and unpublished studies (gray literature) and followed the three-step approach recommended by JBI (Peters et al., 2020). First, an initial limited search of PubMed and Google Scholar was conducted to identify relevant studies and refine search terms. In Google Scholar, the first 100 results sorted by relevance were screened to support term development and capture interdisciplinary literature. Title and abstract terms, along with index terms from relevant records, were analyzed to inform a comprehensive search strategy structured according to the PCC framework (Peters et al., 2020).

The final search string was iteratively refined and then adapted for each information source. Searches were conducted in PubMed, Web of Science, Scopus, and APA PsycINFO, with additional gray literature searches in ProQuest Dissertations and Google Scholar. Full search strategies for all sources are provided in Supplementary Table S5. Reference lists of all included studies were also screened to identify additional relevant sources. The final search was conducted on February 23, 2026.

### 5.2 Source of evidence selection

**Figure 1:**
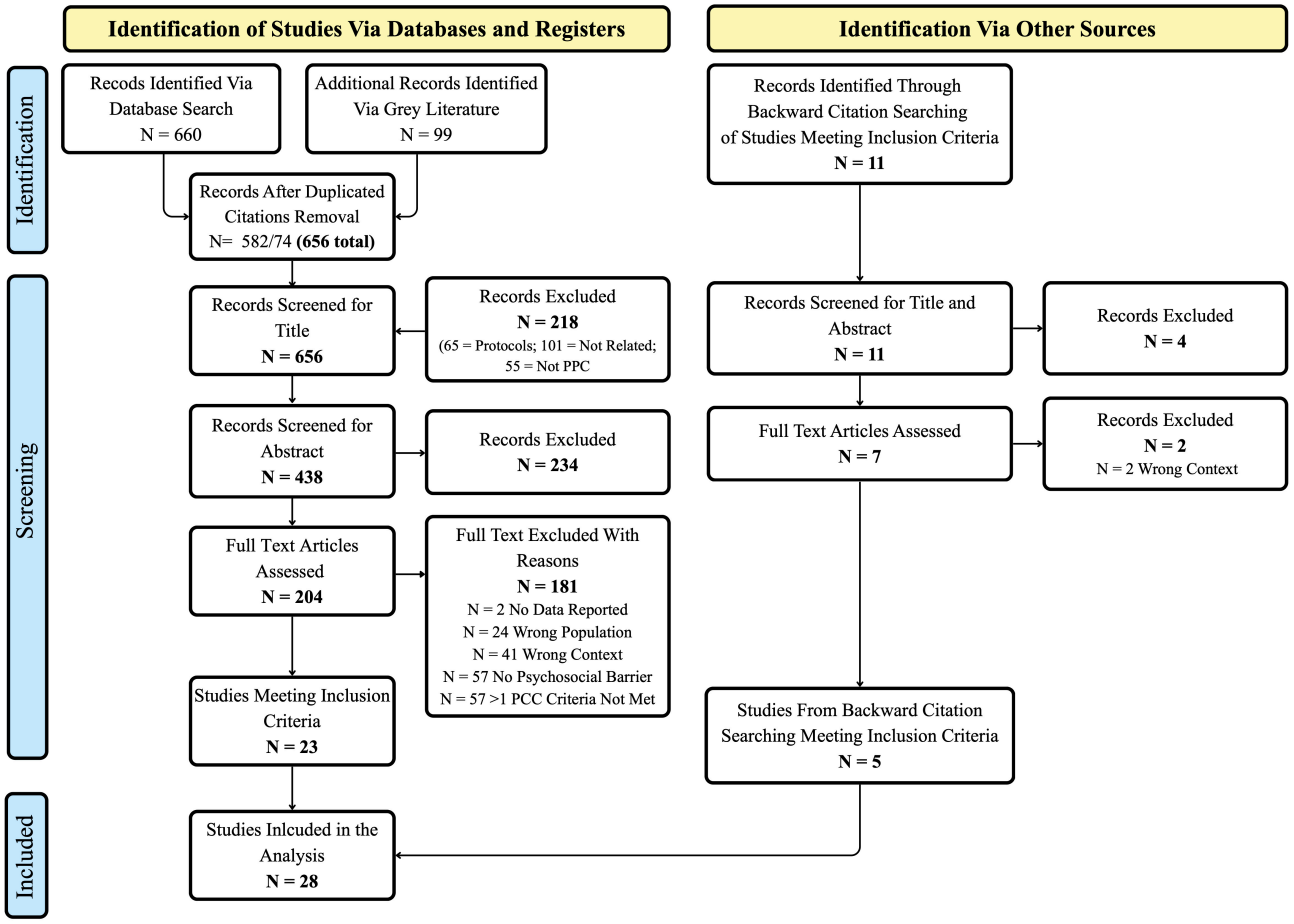
PRISMA flow diagram of the scoping review process.

Following the search, all identified records were imported into Rayyan (Ouzzani et al., 2016) and duplicates were removed. After a pilot screening exercise to ensure consistency, titles and abstracts were independently screened by two reviewers against the predefined inclusion criteria (see Section 3). The full screening procedure is described in Supplementary material (Section 2). Potentially relevant sources were retrieved in full and assessed independently by two reviewers for eligibility. Disagreements at any stage were resolved through discussion and, when necessary, consultation with a third reviewer. The search and selection process is summarized in the PRISMA ScR flow diagram (Tricco et al., 2018) in Figure 1.

### 5.3 Data extraction

Data were extracted by one reviewer using a review-specific data extraction tool developed for this scoping review (Supplementary material, Section 3). Informed by the PCC framework (Peters et al., 2020) and the review objectives, the tool captured study, participant, and intervention characteristics, psychosocial factors, related outcomes, and process indicators. The tool was pilot-tested and refined before full extraction. Critical appraisal was not undertaken because the review aimed to map the evidence base rather than assess methodological quality, consistent with JBI guidance (Peters et al., 2020). Sufficient data were available from the published reports to address the review objectives. Therefore, no contact with study authors was deemed necessary.

Psychosocial factors were classified as barriers when the full text indicated that they limited, discouraged, impeded, or complicated the target behavior or participation in the digital intervention. One *barrier instance* was defined as a distinct psychosocial barrier identified within an individual study. For each instance, the authors' wording describing the psychosocial factor, any definition or description provided, the linked behavior or process, and the supporting evidence (e.g., qualitative quotations, themes, author interpretations, or quantitative findings) were extracted. The unit of counting was the barrier itself rather than the number of times it was mentioned. Repeated references to the same barrier within a study were counted once, even when supported by multiple data sources. Barriers were treated as analytically distinct for synthesis purposes within the same study. Psychosocial factors were extracted when they were either explicitly labeled as barriers by the study authors, including related terms such as obstacle, constraint, or challenge, or implicitly functioned as barriers by negatively affecting a relevant behavior or intervention-related process, such as walking participation, adherence, engagement, or digital tool use.

As an additional quality-assurance procedure during manuscript revision, a second reviewer independently verified the extracted data for a randomly selected sample of 6 of the 28 included studies, corresponding to 21.4% of the final sample. This procedure was not a full re-extraction, but an independent verification of the extracted data against the original full-text articles. The verification focused on key data-charting fields, including study characteristics, intervention characteristics, behavior change components (BCTs), psychosocial barrier identification, barrier classification, and the links between barriers and reported outcomes or intervention processes. Overall, the verification showed high consistency of the extracted data, with 95.1% item-level agreement (137/144). Discrepancies were mainly limited to wording refinements or interpretive classification fields, supporting the stability of the data-charting process. All discrepancies were documented, discussed, and resolved, and minor corrections were incorporated into the supplementary data (Supplementary Table S17).

### 5.4 Data synthesis and presentation

Extracted data were synthesized descriptively and narratively in line with scoping review methodology (Peters et al., 2020). The evidence was mapped to provide an overview of the psychosocial barriers identified, their linked outcomes, and how these barriers were conceptualized and operationalized. Detailed study-level mappings of psychosocial barriers and their linked outcomes are provided in the supplementary data (Supplementary Tables S1–S4) and Supplementary material Supplementary Tables S5–S18).

#### 5.4.1 Psychosocial barriers coding

For each barrier, one primary Capability, Opportunity, Motivation-Behavior (COM-B) model (Michie et al., 2011) and then the Theoretical Domains Framework (TDF) domain (Cane et al., 2012) were assigned based on the dominant mechanism through which the barrier appeared to relate to the relevant behavioral outcome or intervention-related process, consistent with the Behavior Change Wheel (Michie et al., 2011; Cane et al., 2012). This single-domain approach was used to improve transparency, reduce over coding, and support coder agreement (Atkins et al., 2017).

A single best-fit rule was applied because many extracted barriers could plausibly map to more than one COM-B or TDF domain. For example, low confidence in using a digital tool may overlap with capability, beliefs about capability, and motivation. To avoid inflating frequency counts and to maintain comparability across heterogeneous study designs, each barrier was assigned to the domain that best represented its dominant mechanism in the context of the original study. This judgement was based on the study authors' wording, the linked behavior or intervention-related process, and the supporting evidence reported in the full text. Coding was conducted independently by two reviewers, with discrepancies resolved through discussion and, when necessary, consultation with a third reviewer. Inter-rater agreement for the initial coding was assessed using percent agreement and Cohen's kappa in Jamovi (The jamovi project, 2025). The resulting coded instances were then synthesized into reviewer-generated higher-order barrier domains, which were used as descriptive synthesis categories (Supplementary Table S11).

#### 5.4.2 Cross-study harmonization of study characteristics

To enable cross-study comparison and concise reporting across heterogeneous intervention descriptions, key study and intervention characteristics were harmonized into simplified reporting categories using reviewer-defined harmonization rules applied consistently to the original study descriptions.

Because the included studies used heterogeneous terminology and varied reporting formats, the reviewers developed some synthesis categories to enable structured and transparent mapping of the evidence. These categories, including barrier domains and barrier-related outcome groupings, should be interpreted as pragmatic descriptive groupings rather than as mutually exclusive or validated constructs. Some conceptual overlap between categories is expected. Assigning evidence to synthesis categories involved interpretive judgement and represents an analytical simplification used to support evidence mapping. Therefore, frequency counts should be interpreted as counts of how the evidence was organized in this review, rather than as evidence of independent or sharply bounded constructs.

*Participants' age*. Age was extracted at the study level, where reported. When age was presented only by trial arm or study group, group-specific values were retained. For reporting, age was categorized into three groups: adults (≥18 years), older adults (≥65 years), and adults and older adults (studies including both ≥18 and ≥65 years).

*Participants' sex/gender*. Sex/gender data were extracted as reported in the original study and summarized descriptively without further recoding where reporting was inconsistent or incomplete.

*Chronic condition*. Chronic condition data were first extracted verbatim, along with any author-provided definition needed to clarify chronicity. For reporting, conditions were categorized as a single chronic condition when the study focused on one condition, or multiple chronic conditions when the eligibility criteria included two or more chronic conditions.

*Research design*. The original study design reported in each article was first extracted verbatim. Each study was then assigned to one reviewer-categorized design category based on its dominant methodological orientation: (a) experimental study, (b) observational study, (c) qualitative study, (d) mixed-methods study, (e) secondary analysis, or (f) pilot/feasibility study. Studies explicitly described as secondary analyses, mixed-methods, or qualitative were coded accordingly. Among the remaining interventional studies, those primarily designed to evaluate intervention effects were coded as experimental studies, while those focused mainly on feasibility, acceptability, usability, or preliminary testing were coded as pilot/feasibility studies. Non-interventional studies that did not meet criteria for any other category were coded as observational studies.

*Study context*. Study context was categorized descriptively based on recruitment setting and intervention delivery. Contexts were grouped into (a) outpatient/ambulatory care, (b) community or home-based, and (c) hybrid models involving clinical recruitment with remote or home-based delivery. This classification was used to reflect non-hospitalized populations while maintaining pragmatic reporting.

*Intervention characteristics*. Intervention characteristics were categorized descriptively according to the dominant technological configuration and delivery format. Categories included (a) web-based interventions with pedometer use, (b) app-linked wearable interventions, (c) mobile app interventions, and (d) digital interventions combined with human support.

*Intervention techniques and components*. Behavior change techniques (BCTs) were extracted from intervention descriptions and grouped into broader reviewer-defined categories to support consistent descriptive synthesis across heterogeneous reporting. This grouping was informed by the Behavior Change Technique Taxonomy v1 (Michie et al., 2013) and by self-regulation and control theory frameworks (Carver and Scheier, 1982), particularly for recurring elements such as goal setting, self-monitoring, feedback, prompts, and goal review. Where studies explicitly reported BCTs, these were recorded as described by the authors. However, where formal BCT labels were not provided, techniques were identified and grouped by the reviewer based on the intervention content or description. These grouped categories were used as a reporting structure rather than as a formal claim of full taxonomic coding (Supplementary Table S7).

*Level of digital support*. The support model captured whether intervention delivery was autonomous or involved human input and was categorized as (a) fully automated or (b) clinician- or human-supported. Interventions were classified as clinician- or human-supported when clinicians, coaches, researchers, or other staff contributed to delivery, monitoring, feedback, tailoring, or behavioral support.

*Walking-related focus*. Walking focus was first identified when explicitly stated in the article. When this was not stated directly, studies were categorized as walking-focused using a reviewer-defined decision based on whether step goals drive the intervention, engagement metrics were based on walking or if the intervention design revolved around increasing walking or step-based behavior. Furthermore, based on intervention description, walking focus was also classified as primary walking focus when walking was the central behavioral target, or as walking within broader PA focus when walking was addressed as one component of a wider PA target.

*Walking measurement tools* were categorized according to the primary source and type of behavioral measurement. Categories included (a) pedometers, (b) consumer wearable activity trackers, (c) smartphone-based activity tracking, (d) research-grade accelerometry, (e) self-reported PA questionnaires, (f) mixed measurement approaches, (g) unclear or not reported, and (h) not applicable.

*Walking-related target behaviors and goals*. Target behaviors were categorized into three mutually exclusive groups to support comparison across heterogeneous interventions: (a) increase walking, (b) maintain or increase walking, and (c) increase walking and other PA. Categorization was based on the extracted target behavior and goal, intervention focus, and walking-related outcomes.

*Barrier-linked outcomes were charted verbatim for each extracted barrier instance*. Categorized represented reported barrier-related outcome and process links and did not imply that a barrier operated exclusively at one level. In this review, “barrier-linked” refers to outcomes or process indicators that were reported, described, or interpreted in relation to a barrier in the source study and did not imply a causal, predictive, or statistically tested association unless this was explicitly reported by the original authors. Categorization was conducted in two steps. First, each barrier instance was assigned one primary raw outcome category based on the dominant reported or described consequence. Raw categories were (a) walking/PA outcomes; (b) engagement/use; adherence, retention, or participation; (c) acceptability/usability/fit; (d) psychological or emotional response; and (e) broader recovery or self-management outcomes. Second, these raw categories were grouped into three higher-order domains: (a) *behavioral outcomes*, (b) *intervention-process outcomes*, and (c) *psychological and broader self-management outcomes*. This higher-order grouping was informed by implementation outcomes theory and behavior change theory, particularly the Behavior Change Wheel (Michie et al., 2011), to distinguish whether barriers were linked primarily to intervention interaction, behavioral performance, or wider psychosocial consequences.

## 6 Results

The database and gray literature searches resulted in 759 records total before duplicate removal. Of these, 421 were retrieved from PubMed, 17 from APA PsycINFO, 153 from the Web of Science Core Collection, and 69 from Scopus. An additional 99 records were identified through gray literature searching, including 31 from ProQuest Dissertations and Theses Global and 68 from Google Scholar. After duplicate removal, 656 records were screened by title and abstract. In total, 204 full-text articles were assessed for eligibility, of which 181 were excluded. The most frequent reasons for full-text exclusion were failure to meet more than one PCC criterion and absence of psychosocial barriers (*n* = 57 each), followed by wrong context, including lack of a walking focus or absence of a digital intervention context (*n* = 41). Less frequent reasons were wrong population (*n* = 24) and lack of reported data (*n* = 2). An additional five articles were included through backward citation searching. In total, 28 studies were included in the scoping review (Figure 1).

### 6.1 Characteristics of included studies

The 28 included studies were published between 2014 and 2026 and conducted in eight countries, indicating a geographically diverse evidence base (Table 1; Supplementary Table S2).

**Table 1:** Summary of study characteristics (*N* = 28).

References	Country	Study type^b^	Sample size	Chronic condition^a^
Robinson et al. (2024)	United States	Secondary analysis	239	COPD
Golbus et al. (2026)	United States	Observational study	30	HF
Robinson et al. (2020)	United States	Secondary analysis	59	COPD
Gualtieri et al. (2016)	United States	Mixed-method study	10	Multimorbidity
Syrjälä et al. (2021)	Sweden	Qualitative study	15	T2D
Marklund et al. (2021)	Sweden	Qualitative study	16	COPD
Bamonti et al. (2025)	United States	Pilot/feasibility study	5	COPD
Ginossar et al. (2021)	United States	Qualitative study	31	Cancer
Robinson et al. (2019)	United States	Secondary analysis	112	COPD
Bamonti et al. (2023)	United States	Secondary analysis	212	COPD
Roberts et al. (2019)	United Kingdom	Qualitative study	32	Cancer
Blondeel et al. (2025)	Belgium	Experimental study	150	COPD
Wan et al. (2017)	United States	Experimental study	109	COPD
Lambert et al. (2024)	United Kingdom	Qualitative study	26	Multimorbidity
Monteiro-Guerra et al. (2020)	Spain	Qualitative study	14	Cancer
Schrier et al. (2021)	United States	Pilot/feasibility study	24	Cancer
Miropolsky et al. (2020)	United States	Qualitative study	13	Cancer
Khairat et al. (2026)	United States	Mixed-method study	32	Cancer
Bentley et al. (2020)	United Kingdom	Pilot/feasibility study	30	COPD
Doyle et al. (2021)	Ireland and Belgium	Pilot/feasibility study	120	Multimorbidity
Wulfovich et al. (2019)	United States	Mixed-method study	200	Multimorbidity
Okwose et al. (2020)	United Kingdom	Qualitative study	16	HF
Frensham et al. (2018)	Australia	Qualitative study	10	Cancer
Vorrink et al. (2016)	Netherlands	Experimental study	157	COPD
Bartlett et al. (2017)	United Kingdom	Mixed-method study	115	COPD
Nguyen et al. (2017)	Australia	Qualitative study	14	Cancer
Irizarry (2017)	United States	Mixed-method study	85	Multimorbidity
Kaye (2014)	United States	Experimental study	90	Cancer

^*a*^COPD, Chronic obstructive pulmonary disease; HF, Heart failure; T2D, Type 2 diabetes; Multimorbidity, Multiple chronic conditions; Cancer, Cancer survivors; ^*b*^categories as described in the Section 5.

Regarding study design, qualitative studies were the most common (*n* = 10, 35.7%), followed by mixed-method studies (*n* = 5, 17.9%). Experimental studies, pilot and feasibility studies, and secondary analyses each contributed to four studies (14.3%), while observational studies were least common (*n* = 1, 3.6%).

This distribution indicates that the evidence base was weighted toward exploratory and process-oriented research, with a smaller proportion of controlled experimental work. This may suggest that the field is still in a developmental phase, prioritizing the identification, conceptualization, and contextualization of psychosocial barriers over hypothesis testing or causal inference. The reliance on qualitative evidence may also explain the variability in how barriers are defined and reported, often embedded within themes, narratives, or process evaluations rather than standardized constructs. The relatively limited number of controlled experimental studies constrains the ability to establish consistent or causal relationships between barriers and behavioral or engagement outcomes.

Most studies included both adults (≥18) and older adults (≥65; *n* = 24, 85.7%), while fewer focused exclusively on adults (*n* = 3, 10.7%) or specifically on older adults (*n* = 1, 3.6%; Table 2). Age was reported inconsistently across studies, with inconsistent use of means, medians, and ranges, precluding the derivation of a comparable central tendency. Consequently, the ability to examine or interpret age-related psychosocial barriers across studies is limited.

**Table 2:** Study characteristics synthesis (*N* = 28).

Study characteristics	Domain	*n* (%)
Study design^a^	Qualitative studies	10 (35.7)
	Mixed-methods studies	5 (17.9)
	Experimental studies	4 (14.3)
	Pilot/feasibility studies	4 (14.3)
	Secondary analyses	4 (14.3)
	Observational studies	1 (3.6)
Population (age group)^b^	Adults (≥18) and older adults (≥65)	24 (85.7)
	Adults (≥18) only	3 (10.7)
	Older adults (≥65) only	1 (3.6)
Sample size^c^	Median [range]	31.5 [5–239]
Sex and gender reporting	Reported	26 (92.9)
	Not reported	2 (7.1)
Sex and gender distribution (*n* = 26)	Female-predominant or exclusively female	13 (50.0)
	Male-predominant or exclusively male	8 (30.8)
	Approximately balanced	4 (15.4)
	Nonbinary reported	1 (3.8)
Chronic condition^d^	COPD	11 (39.3)
	Cancer survivors	9 (32.1)
	Multiple chronic conditions	5 (17.9)
	Heart failure	2 (7.1)
	Type 2 diabetes	1 (3.6)

For ^*a*^Study design, ^*b*^population grouping, ^*c*^sex/gender distribution; ^*d*^chronic condition categorization procedures are described in the Section 5.

Sample size varied substantially, ranging from 5 to 239 participants (median = 31.5), indicating considerable heterogeneity in study scale and statistical power (Table 2). This suggests that many studies were small-scale, feasibility-oriented, or exploratory.

Sex and gender distribution was reported in 26 of the 28 included studies. Among those reporting these data, samples were more often female-predominant or exclusively female (*n* = 13, 50.0%) than male-predominant or exclusively male (*n* = 8, 30.8%), while a smaller number of studies reported approximately balanced distributions (*n* = 4, 15.4%). One study reported a nonbinary participant (*n* = 1, 3.8%; Table 2).

Regarding chronic conditions, chronic obstructive pulmonary disease (COPD) was the most frequently represented condition (*n* = 11, 39.3%), followed by cancer survivors (*n* = 9, 32.1%). Fewer studies included individuals with multiple chronic conditions, such as combinations of overweight or obesity, hypertension, type 2 diabetes, hyperlipidemia, joint pain, low mood, and other comorbidities (*n* = 5, 17.9%). Heart failure was less commonly represented (*n* = 2, 7.1%), while type 2 diabetes appeared in only one study (*n* = 1, 3.6%). One study (Bartlett et al., 2017) included a mixed population: in the interview arm, people with COPD were studied together with careers and healthcare professionals, while the questionnaire arm included only people with COPD. This study was retained because results for the chronic condition sample could be distinguished from those of the mixed population (Table 2).

From an interpretive perspective, the evidence base is focused on a limited range of clinical conditions, particularly COPD and cancer populations. Consequently, the observed patterns may have limited transferability to less represented chronic conditions and may not fully capture psychosocial barriers associated with different illness identities, self-management demands, prognostic concerns, or treatment contexts.

Taken together, these results indicate that psychosocial barriers in digital walking interventions have been primarily studied through a process-focused, context-sensitive lens, with strong emphasis on user experience and intervention interaction. While this supports rich conceptual development, it also means that evidence linking barriers to outcomes remains uneven and often indirect.

Regarding intervention type, the most common format was digital interventions combined with human support (*n* = 11, 39.3%), followed by web-based interventions combined with pedometers (*n* = 8, 28.6%). The least represented approaches included app-wearable combinations (*n* = 6, 21.4%) and mobile applications alone (*n* = 3, 10.7%). In terms of digital support level, interventions were equally divided between clinician- or human-supported and fully automated delivery models (*n* = 13, 46.4% each), while a small number of studies could not be classified (*n* = 2, 7.1%; Supplementary Table S6).

In most studies, walking was the primary focus of the intervention (*n* = 18, 64.3%), while a smaller proportion embedded walking within a broader PA focus (*n* = 10, 35.7%). Regarding walking-related behavior or goals, most studies aimed to increase walking (*n* = 21, 75.0%). A smaller number targeted maintenance or increase of walking (*n* = 4, 14.3%) or aimed to increase walking alongside other forms of PA (*n* = 3, 10.7%). For walking measurement, consumer wearable activity trackers were the most frequently used tools (*n* = 9, 32.1%), followed by mixed measurement approaches (*n* = 8, 28.6%) and pedometers (*n* = 6, 21.4%). Objective monitoring approaches predominated, while self-reported measures, smartphone-based activity tracking and research-grade devices (accelerometers) were used rarely (each *n* = 1, 3.6%; Supplementary Table S6)

Regarding study context, interventions were equally delivered in hybrid settings or outpatient/ambulatory care settings (*n* = 10, 35.7% each), while fewer studies were conducted in community or home-based settings (*n* = 8, 28.6%; Supplementary Table S6).

Overall, the interventions were heterogeneous but showed a patterned evidence base, characterized by a balance between clinician- or human-supported and fully automated delivery, and by the predominance of structured or semi-supervised contexts. Most interventions focused on initiating increases in walking behavior, whereas maintenance and integration within broader PA patterns were less frequently addressed. This suggests that the identified barriers are more likely to reflect challenges related to intervention uptake, engagement, and supported behavior initiation than to long-term maintenance or autonomous behavior change. Measurement approaches were primarily based on consumer-grade wearable technologies, indicating a reliance on pragmatic tools for real-world monitoring, although variability in measurement methods also pointed to limited standardization in outcome assessment across studies.

### 6.2 Intervention techniques and components

Data on intervention techniques and components were extracted by one reviewer and coded into eleven main domains for reporting. Group descriptions and rationale are detailed in Supplementary Tables S7, S8.

Across the included studies, *self-monitoring and feedback* were the most consistently reported behavior change technique category, identified in all interventions for which this information was available (28/28, 100%). These techniques were reported equally often in clinician- or human-supported interventions and fully automated interventions (*n* = 13, 46.4% each). They involved step tracking, progress monitoring, and iterative or automated feedback. This pattern suggests that self-monitoring is a central operational component of digital walking interventions in this evidence base, serving as the core operational backbone.

*Goal setting and planning* were also highly prevalent (*n* = 25 studies, 89.3%) and were reported equally often in clinician- or human-supported interventions and fully automated interventions (*n* = 12, 42.9% each), including step-based goals, graded goals, and action planning strategies. The results indicate that most interventions combined monitoring with explicit behavioral target-setting. This suggests that interventions were designed around self-regulation architecture, where behavioral targets (e.g., step goals) are continuously adjusted based on performance feedback.

Additional BCT categories included *motivational support and reinforcement* (*n* = 17, 60.7%) and social support (*n* = 15, 53.6%), both more frequently reported in fully automated interventions (*n* = 10, 35.7% each).

*Tailoring and personalization* techniques were identified in 42.9% of interventions (*n* = 12) and were more commonly implemented in clinician- or human-supported interventions (*n* = 6, 21.4%).

*Prompts and reminders* were reported in 39.3% of studies (*n* = 11) and were equally distributed across clinician- or human-supported and fully automated interventions (*n* = 5, 17.9% each), typically supporting engagement with self-regulation processes rather than directly targeting behavior in isolation.

Less frequently reported categories included *education and instruction* (*n* = 10, 35.7%), predominantly in fully automated interventions (*n* = 7, 25.0%), as well as *coaching and human support* (*n* = 5, 17.9%) and *problem solving and barrier management* (*n* = 6, 21.4%), both primarily identified in clinician- or human-supported interventions (*n* = 4, 14.3%; *n* = 5, 17.9%, respectively).

**Figure 2:**
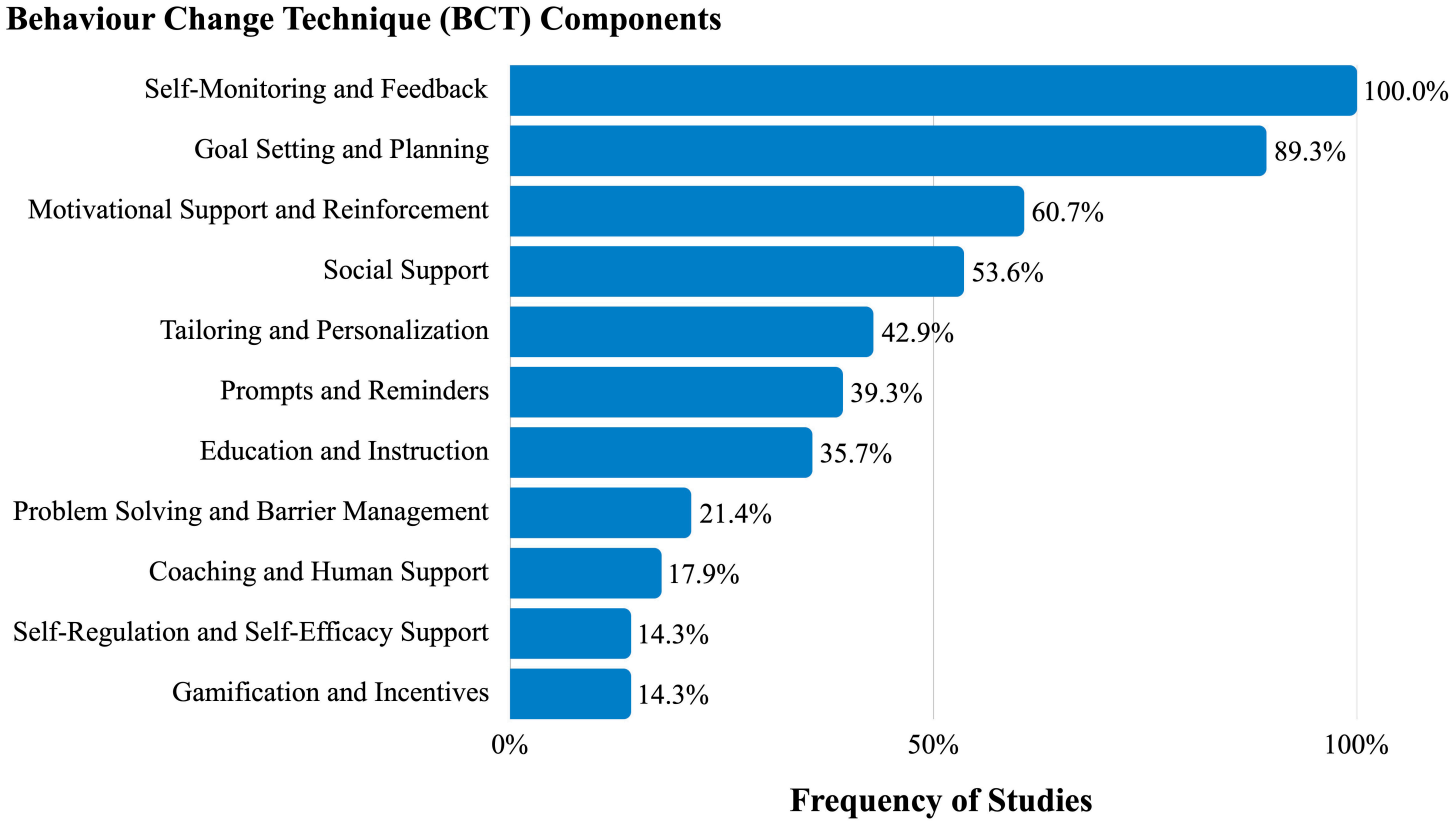
Behavior changes technique (BCT) intervention components (*N* = 28).

*Self-regulation and self-efficacy support*, as well as *gamification*, were the least commonly implemented components (*n* = 4, 14.3%). These showed variation by intervention type, with self-regulation and self-efficacy support more frequently reported in clinician- or human-supported interventions, and gamification more commonly observed in fully automated interventions (*n* = 3, 10.7% each; Figure 2; Supplementary Table S8).

Based on the results, it is suggested that the dominance of self-monitoring and goal setting BCT provides an implicit operationalization of behavior change, where psychosocial barriers are often addressed indirectly (e.g., through feedback or reinforcement) rather than through targeted mechanisms (e.g., addressing beliefs, emotions, or contextual constraints).

Regarding intervention BCT components across levels of digital support and walking target behaviors (Supplementary Table S9), self-monitoring and feedback were concentrated in interventions targeting increased walking, particularly those delivered in fully automated (*n* = 10, 35.7%) and clinician- or human-supported formats (*n* = 9, 32.1%). Goal setting and planning showed a very similar pattern, appearing equally in interventions targeting increased walking delivered via fully automated and clinician- or human-supported models (*n* = 9, 32.1% each). Domains such as motivational support, reinforcement, and social support were used primarily in fully automated interventions targeting increased walking (*n* = 7, 25.0% for each). Tailoring and personalization, prompts and reminders, and education and instruction were moderately represented and showed a more mixed distribution across target behaviors and support formats, although each remained centered on interventions targeting increased walking. Coaching and human support were more concentrated in clinician- and human-supported interventions targeting increased walking (*n* = 3, 10.7%), whereas problem solving and barrier management were largely restricted to the same intervention configuration (*n* = 5, 17.9%). Self-regulation and self-efficacy support also appeared mainly in clinician- or human-supported interventions targeting increased walking (*n* = 3, 10.7%), while gamification and incentives were more often observed in fully automated interventions (*n* = 2, 7.1%) than in clinician-or human-supported formats.

Results regarding BCT, particularly in interventions targeting increased walking, suggest that barrier-linked outcomes (e.g., disengagement, non-adherence) may be more strongly associated with how these core components are experienced (e.g., burden of tracking, misalignment of goals) rather than their mere presence. In addition, core components, such as self-monitoring and feedback, and goal setting and planning, were evenly distributed across fully automated and clinician- or human-supported interventions, indicating that these elements are considered essential regardless of delivery mode. In contrast, components such as coaching, problem solving, and self-efficacy support were more concentrated in clinician-or human-supported formats, suggesting that these strategies may require human facilitation or are preferentially implemented in more resource-intensive intervention models. On the other hand, motivational support, social support, and gamification were more frequently observed in fully automated interventions, reflecting an attempt to simulate or replace human support through digital means.

Overall, this distribution indicates that digital walking interventions are predominantly structured around behavioral regulation mechanisms, with comparatively less emphasis on components explicitly designed to address psychosocial barriers or to support emotional and contextual adaptation. The limited integration of barrier management strategies suggests that intervention design has not yet fully operationalized the psychosocial barriers identified in the literature, reinforcing the gap between barrier conceptualization and intervention implementation.

### 6.3 Review findings

Across the 28 included studies, 105 verbatim psychosocial barrier instances were identified and grouped into seven review-driven higher-order domains, as described in Table 4. For synthesis, a separate barrier instance was defined as a distinct psychosocial barrier identified within an individual study (see Section 5; Supplementary Table S3). Inter-rater agreement was assessed using unweighted Cohen's kappa (Table 3). Agreement was 68% (κ = 0.621), indicating substantial agreement.

**Table 3:** Interrater reliability for barrier coding (*N* = 105).

Method	Cohen's kappa for two raters
Subjects	105
Raters	2
Agreement %	68
Kappa	0.621
*z*	15.6
*p*-value	< 0.001

**Table 4:** Higher-order reviewer-generated domains of barriers (*N* = 105).

Barrier domain	*N* of studies	*N* of author labels	Example of author labels
Belief-based barriers	15	22	Low perceived value; skepticism toward tracker use; negative beliefs about walking ability.
Digital capability and usability barriers	9	11	Technology-use difficulty; low IT confidence; low digital self-efficacy or limited digital literacy; low technology self-efficacy.
Emotional barriers	15	22	Fear of physical activity; technology frustration; shame, guilt; fear or aversion to information; perceived monitoring stress; anxiety/fear related to physical activity or disease.
Motivation-related barriers	10	11	Lower technology engagement; amotivation; controlled motivation; low motivation; declining self-monitoring motivation.
Self-regulation and goal-management barriers	10	16	Perceived unattainable step goals; low confidence in reaching walking goals; low self-efficacy.
Social and relational barriers	10	12	Low ongoing support; fear of negative evaluation by co-workers; low social support or need for peer support; negative peer interaction.
Support and contextual resource barriers	10	11	Perceived lack of time; choice confusion; limited caregiver support; distrust in device data; low professional support.

*N* of author labels indicate verbatim psychosocial barrier instances (*n* = 105).

Across the extracted dataset, 105 coded barrier instances were mapped to seven higher-order barrier groups (Table 4; Supplementary Table S11). The higher-order barrier domains represent reviewer-generated descriptive synthesis categories derived from primary COM-B and TDF coding (see Section 5; Michie et al., 2011, 2005).

The most prominent groups were belief-based barriers (22 instances, 21.0%; 15 studies) and emotional barriers (22 instances, 21.0%; 15 studies), followed by self-regulation and goal-management barriers (16 instances, 15.2%; 10 studies). Social and relational barriers accounted for 12 instances (11.4%; 10 studies), while motivation-related barriers (11 instances, 10.5%; 10 studies), support and contextual resource barriers (11 instances, 10.5%; 10 studies), and digital capability and usability barriers (11 instances, 10.5%; 9 studies) were represented at similar levels (Table 4).

Belief-based and emotional barriers emerged as the most prominent domains, together accounting for 44 of 105 (42%) identified instances. Belief-based barriers primarily involved negative cognitive appraisals related to both the behavior (e.g., walking ability) and the intervention (e.g., perceived value or usefulness of digital tools), indicating that expectancy-value mechanisms and outcome beliefs are central constraints on engagement.

Emotional barriers (e.g., fear, frustration, and monitoring-related stress) reflected affective responses that may directly inhibit behavioral initiation or persistence, suggesting a proximal influence on walking behavior and interaction with the intervention. This domain also included illness-related responses when the dominant mechanism was affective rather than purely clinical or physical. For example, fear of shortness of breath in COPD (Bamonti et al., 2025) was closely reported in relation to the somatic illness experience, but was coded as Motivation under COM-B and Emotion under TDF because the barrier primarily reflected fear and anticipated discomfort rather than physical limitation alone. This suggests that illness-related emotional barriers, such as fear of symptom exacerbation, breathlessness, or bodily discomfort, may be underreported in the current evidence base. The limited number of such examples may reflect a reporting gap, whereby studies document illness-related difficulties mainly as physical or clinical constraints, while the emotional processes attached to these experiences are less frequently identified as distinct psychosocial barriers. Consequently, illness-related emotional barriers may be underrepresented or insufficiently differentiated in studies of digital walking interventions.

Self-regulation and goal-management barriers were characterized by difficulties in setting, monitoring, or achieving goals (e.g., perceived unattainable step targets, low confidence), pointing to breakdowns in self-regulatory processes such as goal calibration, feedback integration, and behavioral control.

Mid-range representation was observed for social and relational, motivation-related, support and contextual resource, and digital capability and usability barriers, each contributing comparably to the overall distribution. Social and relational barriers included limitations in interpersonal support and social dynamics (e.g., lack of encouragement, negative evaluation), indicating that social opportunity moderates both behavioral engagement and sustained participation.

Motivation-related barriers reflected deficits in both intrinsic and extrinsic motivational drivers (e.g., amotivation, declining engagement), suggesting variability in motivational regulation over time.

Support and contextual resource barriers captured external constraints such as time availability, caregiver or professional support, and trust in system outputs, highlighting the importance of environmental and organizational conditions for intervention uptake.

Digital capability and usability barriers reflected limitations in users' skills and confidence in interacting with technology (e.g., low digital literacy, usability challenges), indicating that psychological and physical capability to use digital tools remains a critical precondition for effective engagement.

The distribution of barrier domains indicates that cognitive-affective mechanisms (belief-based and emotional) and self-regulatory processes are the dominant sources of friction within digital walking interventions, whereas contextual, social, and capability-related factors function as enabling or constraining conditions that shape how individuals interact with intervention components. This pattern supports a multi-level interpretation of barriers, where individual-level determinants (beliefs, emotions, and self-regulation) are reported in relation to behavior, while interactional and contextual factors (digital capability, support, and social environment) described as relevant to engagement and implementation processes.

#### 6.3.1 Barrier conceptualization

Explicit barriers, described in direct, problem-oriented terms such as barriers, obstacles, challenges, or limitations, were slightly more common than implicit ones (59/105, 56.2%; Supplementary Table S10). This suggests that most studies framed psychosocial barriers as practical impediments to intervention participation or walking behavior.

Authors also used varied terms for conceptually similar constructs, indicating conceptual overlap but inconsistent labeling across studies. This inconsistency may help explain why psychosocial barriers are not systematically or explicitly integrated into digital walking interventions.

**Figure 3:**
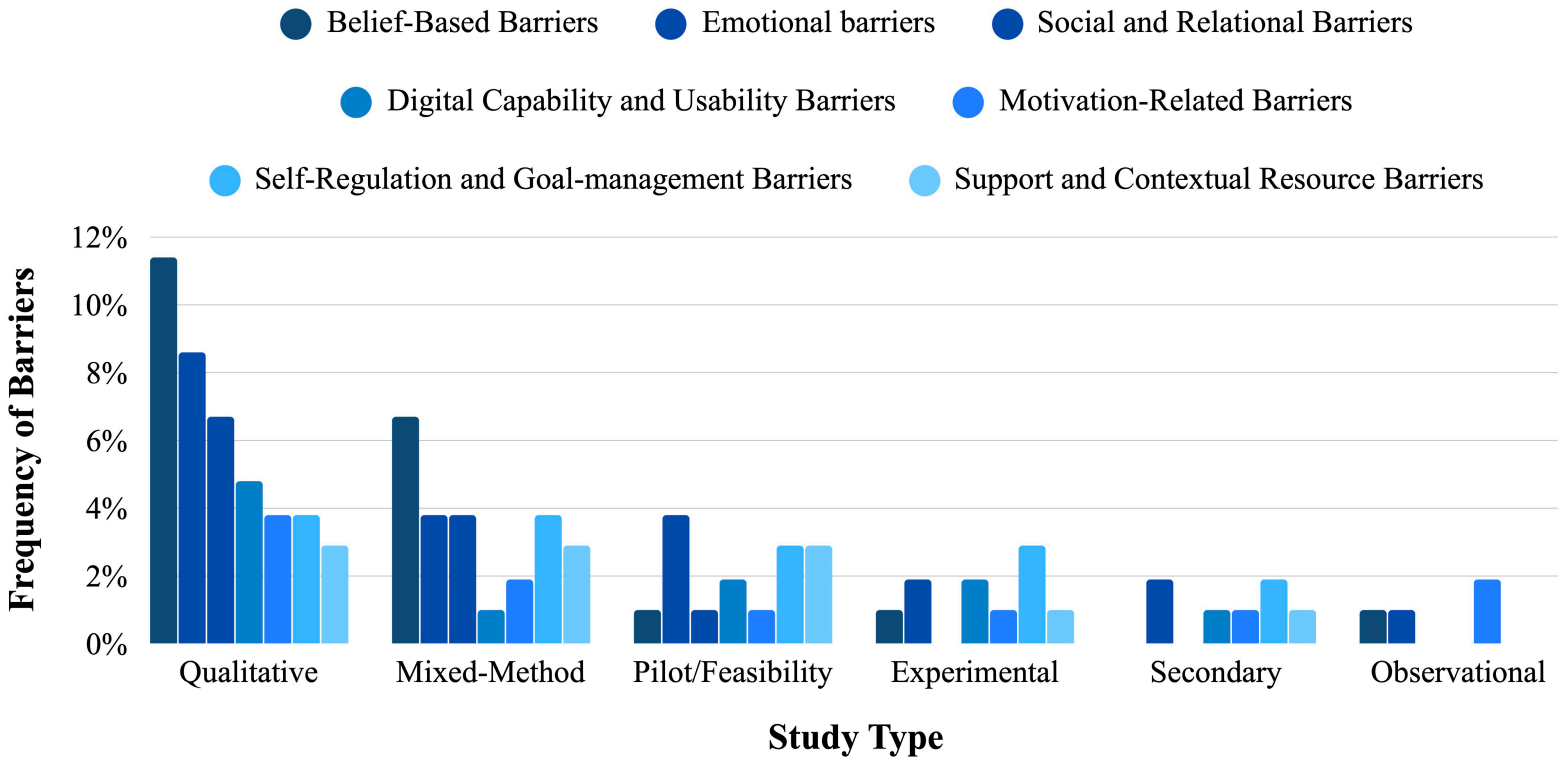
Barrier domains by study type (*N* = 105).

When examined by study type (see Section 5; Supplementary Table S12), barrier domains were most frequently represented in qualitative studies, particularly for belief-based (*n* = 12, 11.4%), emotional (*n* = 9, 8.6%), and social and relational barriers (*n* = 7, 6.7%). Mixed-methods studies also contributed substantially, especially to belief-based barriers (*n* = 7, 6.7%) and to emotional, self-regulation and goal-management, and social and relational barriers (each *n* = 4, 3.8%). By contrast, experimental studies contributed fewer barrier instances overall, with the highest representation for self-regulation and goal-management barriers (*n* = 3, 2.9%), followed by digital capability and usability and emotional barriers (each *n* = 2, 1.9%). Pilot and feasibility studies showed a similar pattern, with emotional (*n* = 4, 3.8%), self-regulation and goal-management, and support and contextual resource barriers each contributing (*n* = 3, 2.9%). Observational studies were represented mainly by motivation-related barriers (*n* = 2, 1.9%; Figure 3).

The pattern indicates that psychosocial barriers were primarily identified through exploratory study designs, rather than being consistently operationalized in experimental or observational studies. Psychosocial barriers were assessed using heterogeneous methods (Supplementary Table S10). Most instances were derived from qualitative interviews or focus groups (*n* = 70, 66.7%), while structured quantitative methods, including validated psychometric scales (*n* = 12, 11.4%), were used less frequently. Other approaches, such as free-text responses, study-specific items, and mixed-methods findings, contributed only marginally.

Across barrier domains, qualitative interview and focus-groups methods predominated for majority of them: belief-based (*n* = 17, 16.2%), emotional (*n* = 16, 15.2%), socio-relational barriers (*n* = 9, 8.6%), digital capability and usability (*n* = 7, 6.7%), as well as support and contextual resources (*n* = 8, 7.6%) barriers (Supplementary Table S13). Quantitative assessments were applied inconsistently and to a limited subset of constructs. Psychometric scales were used mainly for emotional barriers (*n* = 4, 3.8%), belief-based barriers (*n* = 3, 2.9%), and self-regulation and goal-management barriers (*n* = 3, 2.9%). Study-specific self-report items were used infrequently, most notably for self-regulation-related barriers (*n* = 2, 1.9%). Mixed-methods integrated findings were also limited, observed primarily in self-regulation and goal-management barriers (*n* = 3, 2.9%) and, to a lesser extent, in belief-based and motivation-related domains.

The results indicate that some psychosocial barriers were treated as measurable psychological constructs, while others were captured as experiential or context-dependent phenomena emerging during intervention delivery. This suggests that many barriers remain under-operationalized and inconsistently measured, highlighting a gap in the standardized assessment of psychosocial constructs across studies.

**Figure 4:**
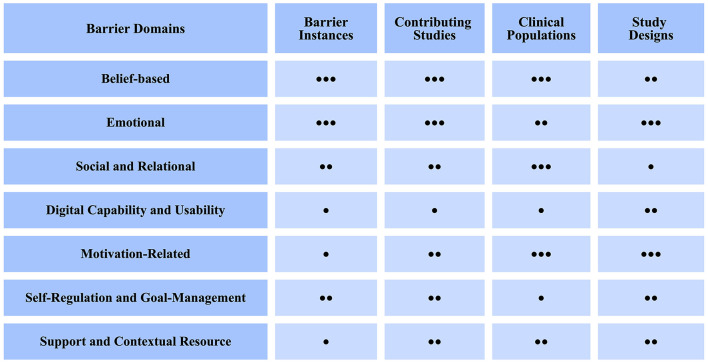
Descriptive evidence distribution across psychosocial barrier domains. Evidence distribution was classified descriptively using thresholds calibrated to the observed range of the revised dataset. One dot indicates low distribution, two dots indicate moderate distribution, and three dots indicate high distribution. For barrier instances, distribution was classified as low (1–11), moderate (12–17), or high (18–22). For contributing studies, distribution was classified as low (1–9), moderate (10–12), or high (13–15). Clinical population representation was classified as low (1–3 categories), moderate (4 categories), or high (5 categories). Study design representation was classified as low (1–3 designs), moderate (4–5 designs), or high (6 designs).

Overall, the distribution of mapped evidence varied across barrier domains (Figure 4; Supplementary Table S18), showing that no domain was supported uniformly across all evidence dimensions. Some domains were supported by broader evidence spread across studies and designs, whereas others were more concentrated within fewer studies, narrower clinical populations, or fewer methodological designs. For example, belief-based and emotional barriers, had a relatively high number of barrier instances and contributing studies, whereas other domains, such as motivation-related barriers or support and contextual resource barriers, were less frequent but still represented across multiple study designs and clinical populations. Although several barrier domains were represented across more than one clinical population, the evidence base was largely driven by studies involving COPD and cancer survivors, with substantially fewer studies involving heart failure, type 2 diabetes, or other chronic conditions. This should be considered when interpreting the psychosocial barrier mapping. Given the modest evidence base, the heterogeneity of chronic conditions, intervention formats, outcome categories, and the generally small study samples, the reported frequency counts should be interpreted as a descriptive synthesis and should not be understood as a prevalence-based or inferential taxonomy, nor as indicators of the relative importance, causal strength, or generalizability of one barrier domain compared to another.

#### 6.3.2 Barrier outcome-related distribution

**Figure 5:**
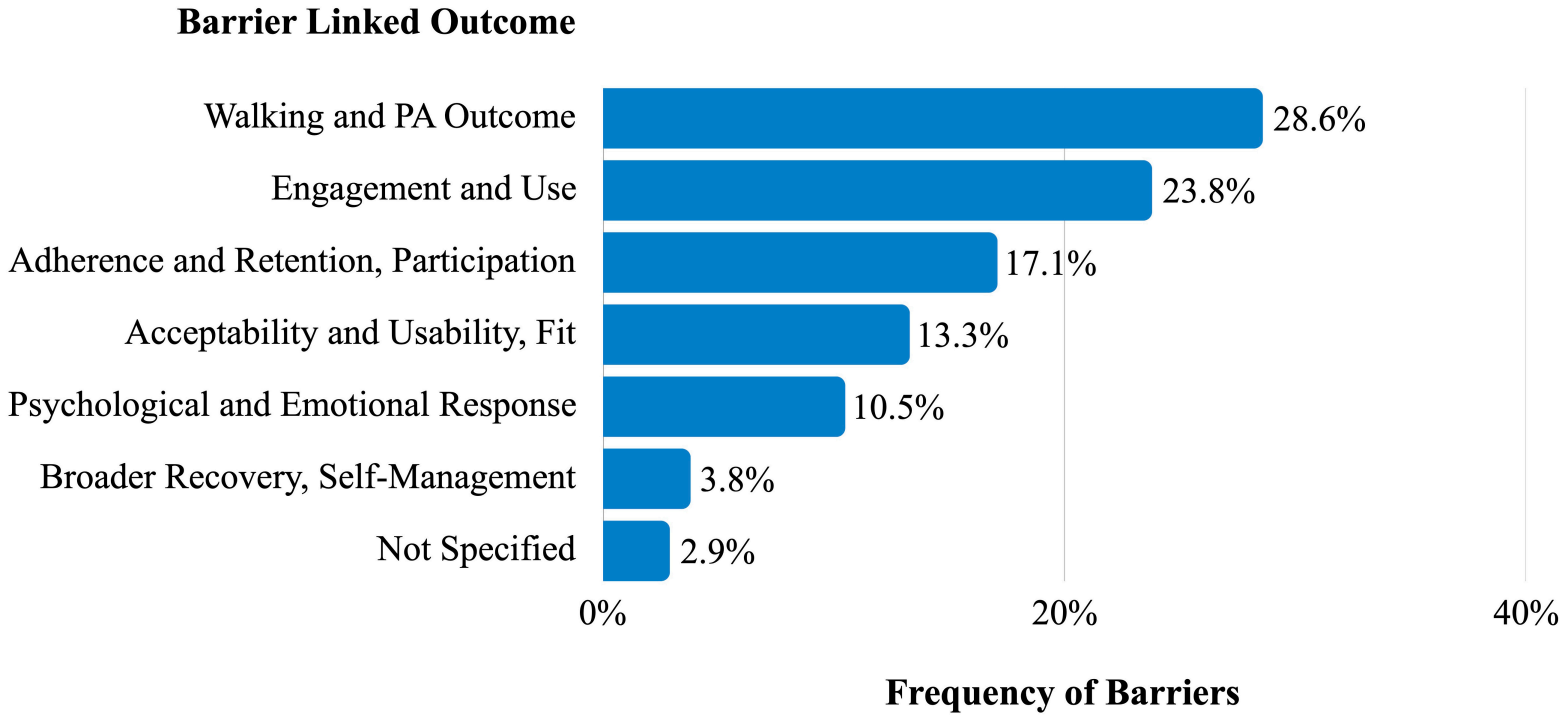
Barriers by detailed outcome groups (*N* = 105).

At the outcome level, the most common linkage was with walking and PA outcomes (*n* = 30, 28.6%), for example lower daily step count, reduced PA participation, and difficulty engaging in home-based walking or activity (Figure 5; Supplementary Table S14). This was followed by engagement and use (*n* = 25, 23.8%), which included lower tool use, eHealth disengagement, and declining engagement over time. Adherence, retention, and participation outcomes accounted for 18 instances (17.1%), including goal attainment difficulty, dropout or trial withdrawal, and lower adherence to physical activity or intervention goals. Acceptability, usability, and fit accounted for 14 instances (13.3%), including reduced intervention credibility, technology acceptance and use, and app acceptability or perceived safety of use. Less frequently reported were psychological and emotional responses (*n* = 11, 10.5%), such as reduced motivation, discouragement, and negative emotional responses to unmet goals, as well as broader recovery, and self-management outcomes (*n* = 4, 3.8%), including broader recovery support needs, need for peer support, and intervention efficacy or ability to benefit. Three instances (2.9%) were not reported in relation to a clearly specified outcome (Figure 5; Supplementary Table S14).

When outcomes were grouped according to their position along the pathway from intervention interaction to behavioral change (see Section 5), the pattern shifted. Intervention-process outcomes were the most common (*n* = 57, 54.3%), followed by behavioral outcomes (*n* = 30, 28.6%), and psychological and broader self-management outcomes (*n* = 15, 14.3%; Supplementary Table S15). These findings suggest that psychosocial barriers have distinct functional roles depending on their position within the behavior change pathway, with outcome linkage varying across domains. Even though barriers tend to cluster around process-related outcomes, indicating that engagement, adherence, and usability are key mediating mechanisms through which psychosocial barriers affect intervention effectiveness (Supplementary Table S15), walking and PA outcomes remain prominent across all barrier incidences (Figure 5; Supplementary Table S14).

**Figure 6:**
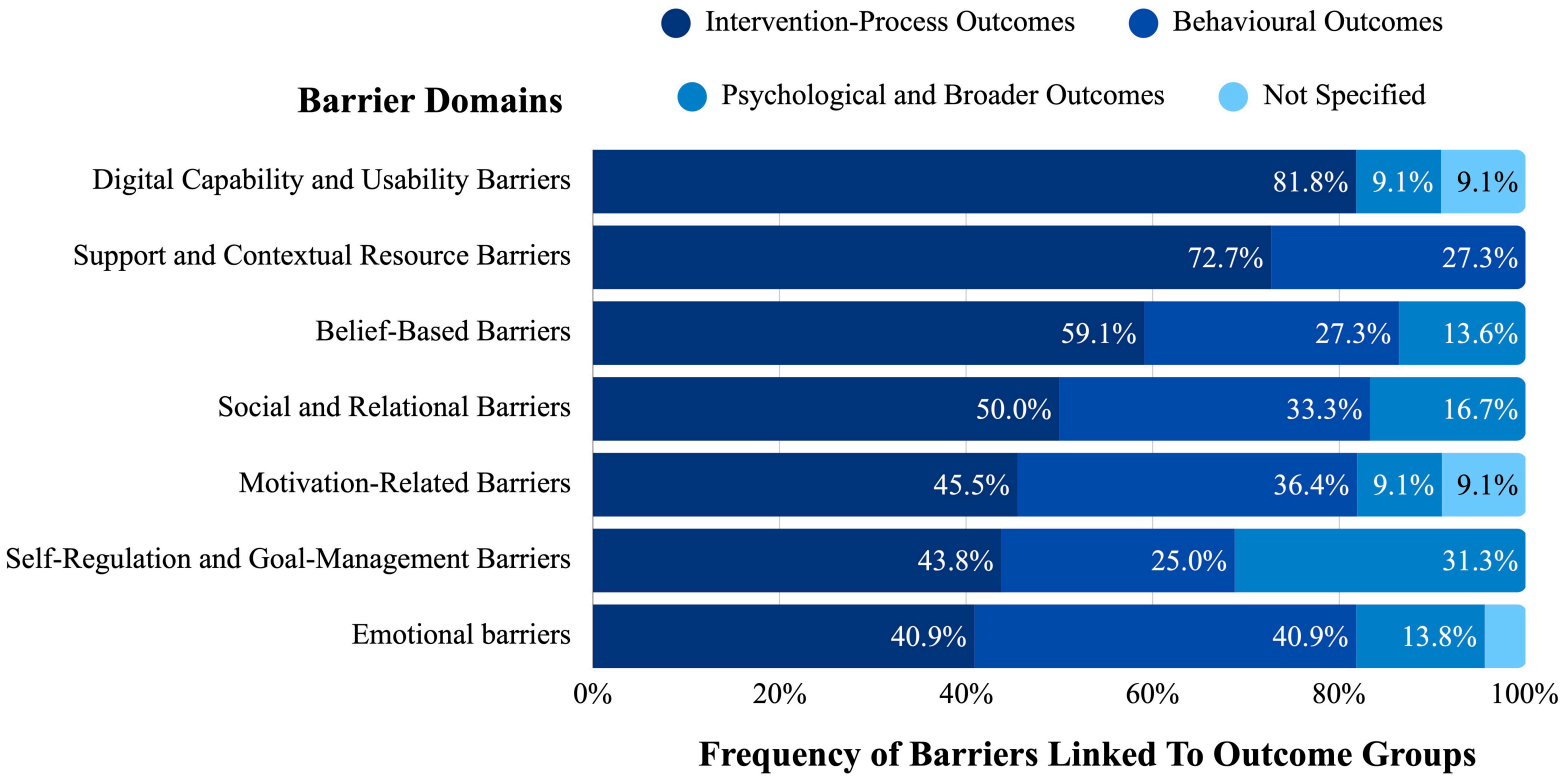
Barrier domains by linked outcome groups.

Intervention-process outcomes were most frequently reported in relation to belief-based barriers (*n* = 13/57, 22.8%), followed by digital capability and usability barriers (*n* = 9/57, 15.8%) and emotional barriers (*n* = 9/57, 15.8%), reflecting reduced engagement, acceptability issues, and non-adherence. Behavioral outcomes were most often associated with emotional (*n* = 9/30, 30.0%) and belief-based barriers (*n* = 6/30, 20.0%). Psychological and broader self-management outcomes were less frequently reported and were primarily reported in relation to self-regulation barriers (*n* = 5/15, 33.3%; Supplementary Table S15). However, within each barrier domain, digital capability-related (*n* = 9/11, 81.8%) and support/context-related (*n* = 8/11, 72.7%) barriers were most closely mapped alongside the intervention process outcomes (Figure 6).

A differentiated pattern was observed among more granular outcomes within each outcome group (Supplementary Table S16). Belief-based barriers were distributed equally across behavioral and intervention-processed outcomes for both walking and PA outcomes (*n* = 6/6, 100%) and engagement/use (*n* = 6/13, 46.2%). Emotional barriers were mainly were reported in relation to behavioral outcomes within walking and PA outcome category (*n* = 9/9, 100%), followed by engagement/use category (*n* = 5/9, 55.6%) within intervention-processed outcome group. Self-regulation and goal-management barriers were consistently linked with intervention-process outcomes, specifically adherence (*n* = 5/7, 71.4%) and psychological or emotional responses (*n* = 5/5, 100%). Social and relational barriers were equally mapped alongside behavioral and intervention-processed outcomes by walking and PA outcomes (*n* = 4/4, 100%) and engagement/use (*n* = 4/6, 66.7%). Digital capability and usability barriers were distributed equally across categories within intervention-process outcomes, including engagement, acceptability, and adherence (each *n* = 3/9, 33.3%), with no instances reported in relation to walking or PA outcomes. Support and contextual resource barriers were mapped alongside engagement/use (*n* = 3/8, 37.5%), adherence/participation (*n* = 3/8, 37.5%), and walking and PA outcomes (*n* = 3/3, 100%) in equal proportion. Motivation-related barriers were mainly reported in relation to walking and PA category (*n* = 4/4, 100%), while engagement/use resulted in 3/5 instances (60.0%).

Intervention-processed outcomes were most frequently reported across all barrier domains, particularly for belief-based, digital-related, and emotional barriers with the engagement/use category most presented for the first two. At a more detailed level, barrier domains were often associated with walking and PA outcomes, especially among belief-based, motivational, emotional, social, and support-related barriers. In contrast, digital-related barriers were not reported in relation to walking or PA outcome categories, and self-regulation barriers were more frequently associated with psychological and emotional response outcomes compared to other categories or barrier domains (Supplementary Table S16).

#### 6.3.3 Mapping of psychosocial barriers to outcomes and study design

**Figure 7:**
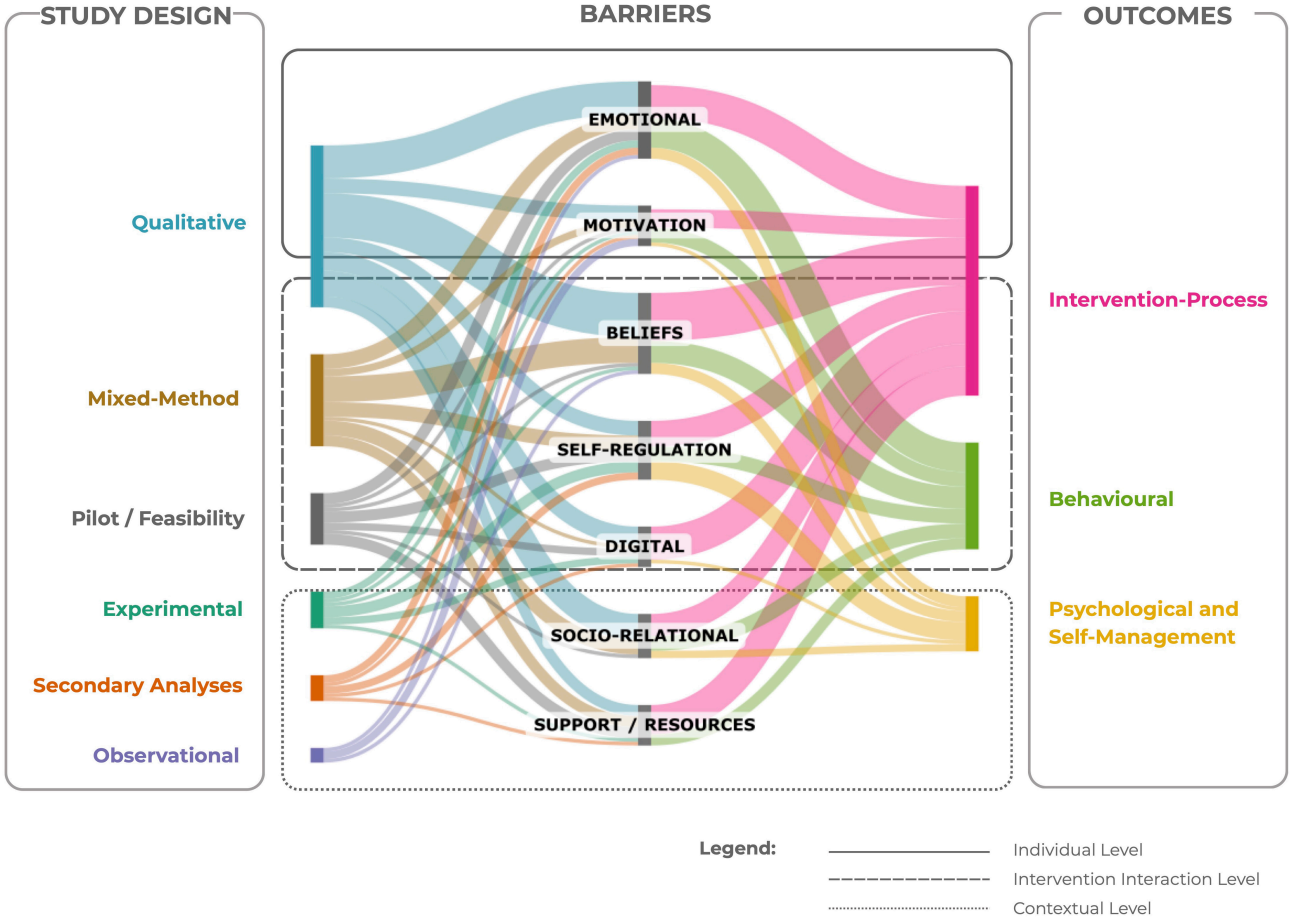
Mapping of psychosocial barrier domains across study design and outcome levels (*N* = 105).

The synthesis of barrier domains across outcome types and study designs revealed a structured but non-uniform pattern in how psychosocial barriers were identified and positioned within the evidence base (Figure 7; Supplementary Table S4).

*Behavioral outcomes, among those reported and extracted*, were most frequently examined in qualitative studies (*n* = 13/30, 43.3%), particularly for social and relational barriers (*n* = 4/12; 33.3%), including lack of social support, limited interpersonal encouragement, and social isolation. Smaller contributions were observed for belief-based barriers (*n* = 3/22; 13.6%), such as low perceived value of the intervention or negative beliefs about walking ability, and emotional barriers (*n* = 3/21; 14.3%), including fear of physical activity, anxiety, or discouragement. Support and contextual resource barriers (*n* = 2/11; 18.2%) and motivation-related barriers (*n* = 1/10; 10.0%), including lack of time, competing demands, or reduced motivation to engage, were least represented.

*Intervention-process outcomes* were the most widely distributed across study designs (*n* = 57), with the largest contributions from qualitative (*n* = 22/57, 38.6%) and mixed-methods studies (*n* = 18/57, 31.6%), and the smallest from experimental and secondary analyses (each *n* = 4/57, 7.0%). Within qualitative studies, belief-based barriers were most frequently examined (*n* = 8; 36.4%), including low perceived relevance, lack of trust, and doubts about effectiveness. In mixed-methods studies, belief-based barriers were also prominent (*n* = 5/22; 22.7%). Digital capability and usability barriers and emotional barriers were most reported in qualitative studies (each *n* = 4/10, 40.0% and *n* = 4/21, 20.0%, respectively), including difficulties using technology, low digital literacy, frustration, and disengagement. In contrast, self-regulation and goal-management (*n* = 4/16; 25.0%) and social and relational barriers (*n* = 3/12; 25.0%) were primarily identified in mixed-methods studies, reflecting challenges in maintaining adherence and engagement. Support and contextual resource barriers were mainly reported in pilot and feasibility studies (*n* = 3/11; 27.3%), including time constraints and environmental limitations. *Psychological and broader self-management outcomes* were least represented (*n* = 15), with the majority identified in qualitative studies (*n* = 7/15, 46.7%). These were most associated with self-regulation and goal-management barriers (*n* = 3/16, 18.8%), including reduced goal confidence, difficulty managing expectations, and challenges sustaining behavior change.

Across all domains, the broadest distribution across outcome types and study designs was observed for emotional barriers, which were represented across all three outcome categories and all six study designs. Motivation-related barriers also showed wide distribution despite lower frequency, likewise appearing across all outcome types and study designs. Belief-based barriers were the most frequent overall and were distributed across all three outcome types and five study designs, followed by self-regulation and goal-management barriers (Supplementary Table S4).

The findings suggest that literature has focused primarily on intervention-process outcomes, particularly within qualitative and mixed-methods designs, while psychological and broader self-management outcomes remain comparatively underexamined.

## 7 Discussion

This scoping review aimed to identify and systematically map psychosocial barriers within digital walking interventions for individuals with chronic illness. Overall, the findings indicate that the evidence base is exploratory, heterogeneous, and primarily process-oriented. Psychosocial barriers were variably conceptualized across studies, which limits comparability but also highlights the need for more consistent approaches to defining, operationalizing, and analyzing barriers in digital walking interventions As a result, existing evidence provides a theory-informed but empirically uneven understanding of how psychosocial barriers are identified, conceptualized, and analyzed in digital walking interventions.

A central finding of this review is the identification of a structured pattern linking psychosocial barriers to three outcome categories: behavioral, intervention-process, and psychological. When outcomes were grouped along the behavior change pathway, intervention-process outcomes were most frequent, followed by behavioral outcomes, with psychological and broader self-management outcomes least represented. However, at a more granular level, walking and PA outcomes constituted the largest single outcome category. This distribution suggests that psychosocial barriers may affect interventions through multiple pathways but are more often reported in relation to engagement-related processes than to walking behavior directly (Sekhon et al., 2017; Yardley et al., 2016). Importantly, when barriers were grouped, the domains representing them showed a cross-level pattern, being mapped alongside more than one outcome category. These findings highlight that psychosocial barriers should not be treated as a homogeneous construct, but rather as functionally distinct mechanisms operating at different points along the behavior change pathway.

It is also possible that the identified psychosocial barriers do not reflect stable patient-level characteristics alone but are partly shaped by intervention features and delivery context. This suggests that some barriers identified in the present synthesis may be responsive to design and implementation choices rather than representing fixed individual constraints. For example, perceived relationships with a healthcare provider or digital coach, trust in the system, and accountability processes may also influence engagement and adherence in digital interventions. Therefore, psychosocial barriers may represent not only targets for assessment, but also modifiable points of intervention within digital walking programme design.

From an implementation perspective, this underscores the importance of considering engagement as a significant design target rather than a secondary outcome, because psychosocial barriers might disrupt the intervention process by limiting user interaction with digital systems. In this sense, engagement may function as a pathway linking intervention delivery to behavioral outcomes. Interventions that fail to address usability, perceived relevance, emotional burden, or contextual constraints may struggle to achieve sustained behavior change, regardless of their theoretical basis (Kelders et al., 2012; Perski et al., 2017; Yardley et al., 2016).

Another key finding is the mismatch between identified barriers and implemented intervention components (BCTs). While belief-based and emotional barriers were most frequently reported, interventions were predominantly structured around only self-monitoring and goal setting techniques. Although these components are well-established within behavior change frameworks, they primarily target behavioral regulation processes and may address psychosocial barriers only indirectly. This suggests an implementation gap, where barriers identified in the review literature are not systematically incorporated into intervention design. As a result, interventions may fail not because BCTs are ineffective, but because users are unable or unwilling to engage with them due to unaddressed psychosocial barriers (Borghouts et al., 2021; Yardley et al., 2016).

### 7.1 Conceptual model of psychosocial barriers

**Figure 8:**
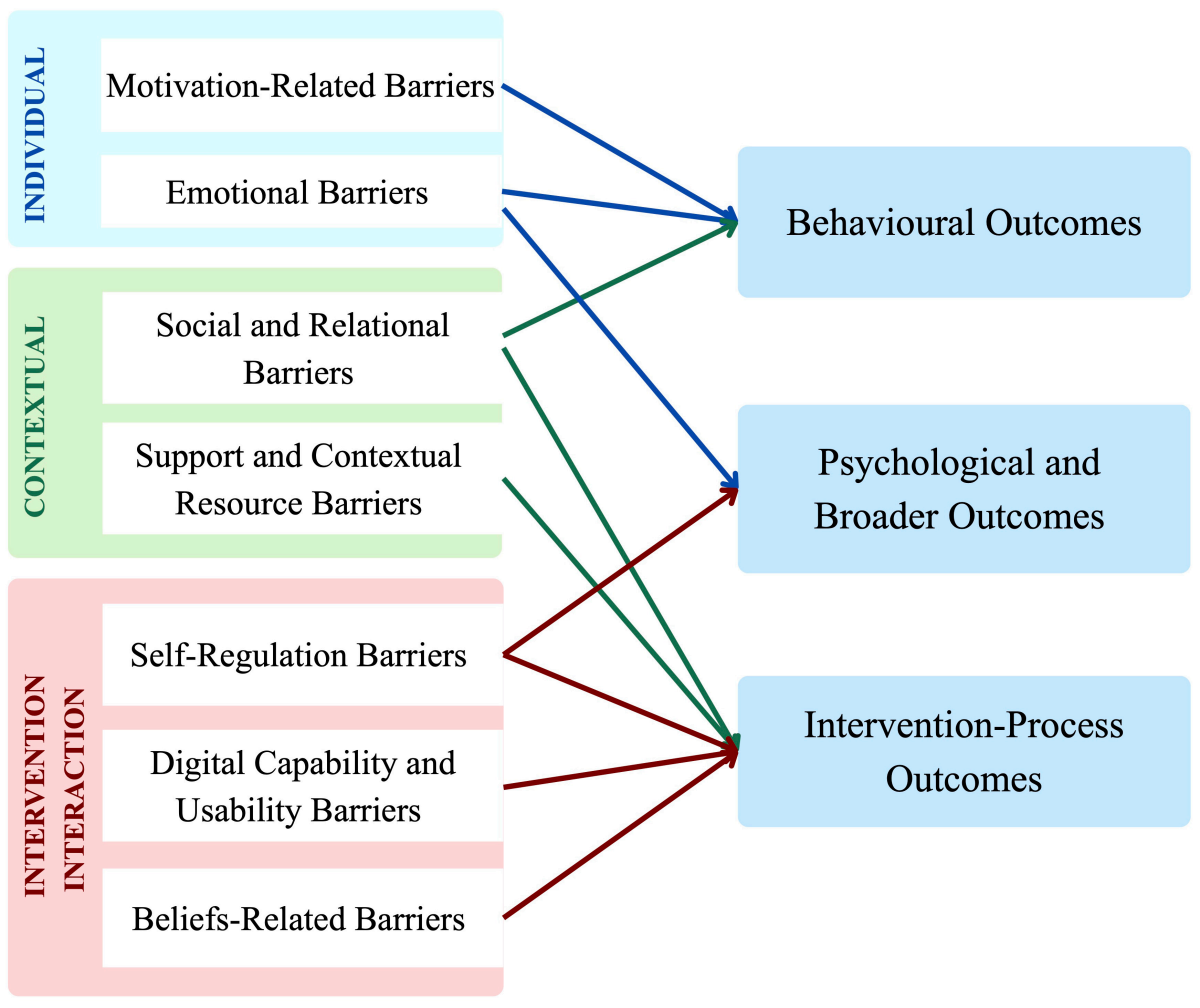
Conceptual model of psychosocial barriers.

Based on the findings of this review, a multilevel conceptual model of psychosocial barriers in digital walking interventions is proposed (Figure 8). The model organizes the identified barrier domains across three interconnected levels: *individual, intervention interaction, and contextual*, reflecting the differential patterns observed in the mapping (Perski et al., 2017; Sallis et al., 2008; Yardley et al., 2016). This model should be interpreted as an author-generated conceptual synthesis of mapped patterns rather than as a tested explanatory or causal model.

Barriers at the individual level, particularly emotional, and motivation-related barriers, were more closely related to behavioral outcomes, suggesting a closer reported linkage with walking and physical activity. In contrast, barriers at the intervention interaction level, including belief-based, digital capability/usability, and self-regulation barriers, were more often reported in relation to intervention-process outcomes such as engagement, adherence, and acceptability. At the contextual level, support- and resource-related barriers were also mainly associated with intervention-process outcomes, particularly sustained participation and integration into daily life. However, social and relational barriers showed a mixed pattern across behavioral and intervention-process outcomes, indicating a cross-level influence. While self-regulation and emotional barriers were also mapped alongside psychological and broader self-management outcomes.

In addition, barriers at the individual level overlapped with (a) the contextual level in relation to behavioral outcomes, indicating that individual barriers may be shaped by broader social and environmental conditions, and (b) the intervention interaction level with shared connections within psychological outcomes, suggesting that experiences of intervention use may affect both engagement and intermediate psychological processes. The contextual and intervention interaction levels also showed overlap within intervention-process outcomes, particularly those related to sustained participation and everyday integration.

Taken together, the results suggest that psychosocial barriers should not be interpreted as isolated factors, but rather as domains that were mapped across interconnected levels with partially overlapping outcome pathways. Furthermore, the findings underscore the need for a more integrated and theoretically grounded approach to psychosocial barriers in digital walking interventions. Rather than treating barriers as isolated factors, future research should consider their dynamic and multi-level nature. More explicit integration of psychosocial barriers into intervention design (particularly those currently under addressed, including social and contextual dimensions) may support the development of more adaptive and sustainable behavior change strategies for chronically ill populations, mainly COPD and cancer survivors.

## 8 Limitations

Several limitations should be considered. Data extraction was conducted by a single reviewer, which may have introduced some risk of selection or extraction bias despite the use of a predefined extraction tool. However, this risk was partly mitigated by a post-review quality check, in which a second reviewer independently verified a random sample of extracted data against the full-text articles, showing high agreement and no substantive changes to the synthesis.

More broadly, the main limitation of this review relates to the heterogeneity of the included literature. The evidence base was dominated by qualitative and small-scale studies, with substantial variation in barrier terminology, assessment methods, and outcome reporting, which limited comparability across studies. In many cases, psychosocial barriers were only briefly described, inconsistently labeled, or insufficiently operationalized, requiring harmonization and categorization by the reviewer to support structured synthesis. Although this approach improved consistency, it may have reduced study-level nuance, and some barriers could plausibly overlap with neighboring domains. Accordingly, the reported categories should be understood as pragmatic groupings generated by reviewer synthesis, and the frequencies as indicative patterns rather than precise estimates of distinct construct prevalence. In addition, the absence of a reported barrier should not be interpreted as evidence of its absence in practice. Generalizability is also limited by the broad age ranges used in many samples, as only one study focused specifically on older adults, and the concentration of evidence in a limited number of chronic conditions, particularly COPD and cancer.

A further limitation is the restriction to English-language publications, which may have introduced language bias and limited the cultural breadth of the synthesis. Although the included studies were conducted across eight countries, this should not be interpreted as comprehensive cultural representation. Psychosocial barriers within digital interventions promoting walking may be shaped by language, culture, healthcare systems, stigma, illness beliefs, and social norms. As a result, barriers reported in non-English-speaking contexts or in studies published in languages other than English may be underrepresented.

Despite these limitations, this review provides a theory-informed synthesis of psychosocial barriers in digital walking interventions for chronic illness, mainly COPD and cancer survivors. By systematically organizing heterogeneous evidence, it highlights patterns in how barriers operate across studies, indicating that psychosocial barriers are multi-level and functionally differentiated. Finally, as this was a scoping review, the aim was to map the evidence rather than assess methodological quality or establish causal relationships. As such, the findings should be interpreted as descriptive rather than inferential (Peters et al., 2020).

## 9 Theoretical and practical implications

The review indicates that engagement should be understood as a mechanism rather than merely a usage metric in digital walking interventions, because many barriers appear to affect outcomes through acceptability, adherence, and sustained use rather than directly through walking behavior alone.

Additionally, some psychosocial barriers may be responsive to how digital walking interventions are designed and delivered, rather than being treated solely as patient-level limitations. This aligns with broader evidence suggesting that key psychosocial processes relevant to engagement (such as confidence in one's ability to perform the behavior, expectations about outcomes, affective responses to technology, and perceived support or trust in the healthcare context) can be influenced by intervention features, including feedback, communication style, and level of human support (Bandura, 1997; Mohr et al., 2011; Bickmore et al., 2005).

Practically, future interventions may benefit from more explicit alignment between psychosocial barriers and selected intervention components. For example, belief-based barriers may require credibility-building, value clarification, or expectancy-focused content, while contextual barriers may require flexibility, practical support, or adaptive tailoring. Together, these findings suggest that addressing engagement-relevant psychosocial processes, rather than focusing solely on behavior regulation may improve sustained use.

## 10 Conclusion

This scoping review shows that psychosocial barriers in digital walking interventions for individuals with chronic illness, such as COPD and cancer survivors, are diverse, multi-level, and unevenly addressed across the current evidence base. The findings indicate that these barriers are linked more often to intervention-process outcomes (engagement, adherence, and acceptability all together), than to walking outcomes directly, suggesting that psychosocial barriers frequently influence intervention effectiveness by disrupting sustained interaction with the digital intervention. Belief-based, emotional and self-regulation barriers emerged as particularly important domains. The literature overall remains dominated by qualitative and process-oriented evidence.

The review suggests that current digital walking interventions rely largely on generic self-regulation techniques, while more specific psychosocial barriers are often identified but not systematically targeted in intervention design. Greater conceptual clarity, more consistent operationalization, and stronger alignment between identified barriers and intervention components are needed to improve sustained engagement and long-term behavioral impact in chronic illness populations, such as COPD and cancer survivors

Greater conceptual clarity and more standardized measurement will be important next steps for strengthening this area of research and improving the real-world sustainability of digital walking interventions.

## Data Availability

The original contributions presented in the study are included in the article/Supplementary material, further inquiries can be directed to the corresponding author/s.
