# Supplementary material for: Exploring psychosocial barriers in digital walking interventions for the chronically ill: a scoping review
Source: Front Psychol. 2026 Jul 17;17:1869579. doi: 10.3389/fpsyg.2026.1869579 (PMC13423769; doi:10.3389/fpsyg.2026.1869579)
Supplement: Supplementary file 2 [file Supplementary_File_2.pdf]

Supplementary Material

SECTION 1. Search strategy

Databases searched:

- PubMed (PubMed)
- APA PsycINFO (ProQuest)
- Scopus (Scopus) - includes grey literature
- Web of science (Web of science) - includes grey literature
- Google scholar (Google scholar) - includes grey literature
- Dissertations & Theses Global (ProQuest) - includes grey literature

Table S5. Initial Search Overview: PCC With Key and Index Words for PubMed

PCC[1]	Criteria	Key and index words
**Population**	Adults (18+) and older adults (65+)	(<br>"Adult"[MeSH Terms]<br>OR "Aged"[MeSH Terms]<br>OR "Aged, 80 and over"[MeSH Terms]<br>OR "Middle Aged"[MeSH Terms]<br>OR "older adult*"[tiab]<br>OR "middle aged"[tiab]<br>OR "middle-aged"[tiab]<br>)
	Chronically ill	(<br>"Chronic Disease"[MeSH Terms]<br>OR "Comorbidity"[MeSH Terms]<br>OR "Multimorbidity"[MeSH Terms]<br>OR "chronic disease*"[tiab]<br>OR "chronic illness*"[tiab]<br>OR "chronic condition*"[tiab]<br>OR "chronic somatic disease*"[tiab]<br>OR "somatic chronic disease*"[tiab]<br>OR cardiovascular condition*<br>OR respiratory condition*<br>OR metabolic condition*<br>OR neurological condition*<br>OR cancer survivor*<br>OR "long-term condition*"[tiab]<br>OR "long term condition*"<br>)
**Concept**	Psychosocial barriers	"Attitude to Health"[MeSH Terms]<br>OR "Motivation"[MeSH Terms]<br>OR "Perception"[MeSH Terms]<br>OR "Self Efficacy"[MeSH Terms]<br>OR "Social Support"[MeSH Terms]<br>OR "Social Isolation"[MeSH Terms]<br>OR "Social Participation"[MeSH Terms]<br>OR "Loneliness"[MeSH Terms]<br>OR "Health Belief Model"[MeSH Terms]<br>OR "Social Facilitation"[MeSH Terms]<br>OR "psychosocial"[tiab]<br>OR "psycho-social"[tiab]<br>OR "social-psychological"[tiab]<br>OR "psychological"[tiab]<br>OR "barrier*"[tiab]<br>OR "difficulty*"[tiab]

		OR "obstacle*"[tiab]<br>OR "facilitator*"[tiab]<br>OR "challenge*"[tiab]<br>OR "attitude*"[tiab]<br>OR "belief*"[tiab]<br>OR "motivator*"[tiab]<br>OR "motivational"[tiab]<br>OR "motivation"[tiab]<br>OR "driver*"[tiab]<br>OR "perception*"[tiab]<br>OR "perceived"[tiab]<br>OR "self-efficacy"[tiab]<br>OR "self efficacy"[tiab]<br>OR "selfefficacy"[tiab]<br>OR "perceived behavioral control"[tiab]<br>OR "perceived behavioural control"[tiab]<br>OR "control belief*"[tiab]<br>OR "experience*"[tiab]<br>OR "preference*"[tiab]<br>OR "social support"[tiab]<br>OR "lack of social support"[tiab]<br>OR "social norm*"[tiab]<br>OR "subjective norm*"[tiab]<br>OR "social isolation"[tiab]<br>OR "socially isolated"[tiab]<br>OR "social disconnection"[tiab]<br>OR "social participation"[tiab]<br>OR "social involvement"[tiab]<br>OR "social engagement"[tiab]<br>OR "loneliness"[tiab]<br>OR "lonely"[tiab]<br>OR "social network size"[tiab]<br>OR "social network support"[tiab]<br>OR "social network ties"[tiab]<br>OR "social network structure"[tiab]<br>OR "interpersonal relation*"[tiab]<br>OR "interpersonal relationship*"[tiab]<br>OR "interpersonal interaction*"[tiab]<br>OR "stigma"[tiab]<br>OR "self-stigma"[tiab]<br>OR "self stigma"[tiab]<br>OR "age stereotype*"[tiab]<br>OR "illness stereotype*"[tiab]<br>OR "intention*"[tiab]<br>OR "stereotype"[tiab]<br>)
**Context**	Digital intervention	(<br>"Mobile Applications"[MeSH Terms]<br>OR "Internet-Based Intervention"[MeSH Terms]<br>OR "Smartphone"[MeSH Terms]<br>OR "Text Messaging"[MeSH Terms]<br>OR "Cell Phone"[MeSH Terms]<br>OR "mobile app*"[tiab]<br>OR "mobile application*"[tiab]<br>OR "mhealth"[tiab]<br>OR "mobile health"[tiab]<br>OR "mobile-based"[tiab]<br>OR "mobile based"[tiab]<br>OR "mobile intervention*"[tiab]<br>OR "ehealth"[tiab]<br>OR "digital health"[tiab]<br>OR "web-based"[tiab]<br>OR "web based"[tiab]

		OR "internet-based"[tiab]<br>OR "internet based"[tiab]<br>OR "web platform*"[tiab]<br>OR "web-based platform*"[tiab]<br>OR "smartphone app*"[tiab]<br>OR "smartphone application*"[tiab]<br>OR "app-based"[tiab]<br>OR "internet-mediated"[tiab]<br>OR "internet mediated"[tiab]<br>OR "mobile technolog*"[tiab]<br>OR "digital therapeutic*"[tiab]<br>OR "digital therap*»[tiab]<br>OR "digital intervention*"[tiab]<br>OR "DTx"[tiab]<br>OR "text message*"[tiab]<br>OR "short message service*"[tiab]<br>OR "SMS"[tiab]<br>OR "behavior change technolog*"[tiab]<br>)
	Physical activity with focus on walking	(<br>"Wearable Electronic Devices"[MeSH Terms]<br>OR "Walking"[MeSH Terms]<br>OR physical activity [MeSH Terms]<br>OR physical inactivity [Mesh Terms]<br>OR "wearable*"[tiab]<br>OR "wearable device*"[tiab]<br>OR "wearable activity tracker*"[tiab]<br>OR "activity tracker*"[tiab]<br>OR "smartwatch"[tiab]<br>OR "walking"[tiab]<br>OR "walk"[tiab]<br>OR "walking intervention*"[tiab]<br>OR "step count*"[tiab]<br>OR "daily step*"[tiab]<br>OR "pedometer*"[tiab]<br>OR "Fitbit*"[tiab]<br>OR "step monitoring"[tiab]<br>)

Note. PCC – Population, Concept, Context;

SECTION 2. Screening tool

Review Objective: to identify and systematically map the existing evidence on psychosocial barriers in digital physical activity interventions promoting walking among chronically ill.

Main Definitions

Adults: individuals aged 18 years and older.

Older adults: individuals aged 65 years and older.

Chronically ill: individuals diagnosed with at least one chronic condition, including but not limited to cardiovascular, pulmonary, metabolic, musculoskeletal, or neurological conditions, as defined by study authors.

Psychosocial barriers: psychological and social factors that may hinder walking behavior or engagement with digital interventions, including but not limited to motivation, self-efficacy, identity, age- or illness-related stereotypes, stigma, perceived social norms, beliefs, and attitudes.

Digital walking interventions: interventions delivered through digital technologies (e.g. mobile applications, wearable devices, web-based platforms, SMS-based programs) aimed at promoting walking.

Overview of inclusion/exclusion criteria

Type of source

Include:

- Journal articles
- Conference papers or outputs (e.g., full conference papers, conference proceedings, abstracts reporting empirical data (if sufficient detail is provided))
- Book chapters
- Reports
- Reviews, such as systematic reviews and meta-analyses that meet the inclusion criteria, were considered only for supplementary searching by screening their reference lists to identify additional relevant primary studies.
- Grey literature
- Studies published in English are included.

Exclude:

- Editorials
- Commentaries
- Opinion papers
- Study protocols without results
- Theoretical papers
- Purely technical or engineering papers without user-level psychosocial data.

- Publications in other than English language are excluded.

Population

Include:

Adults aged ≥18 years; Older adults aged ≥65 years; Individuals diagnosed with at least one chronic condition (e.g. cardiovascular, pulmonary, metabolic, neurological, musculoskeletal; including multimorbidity).

Exclude:

Studies focusing exclusively on children or adolescents;
Healthy populations with no reported chronic condition;
Institutionalized populations only (e.g. nursing homes, long-term residential care), unless combined with outpatient samples;
Studies where results for chronically ill adults cannot be disaggregated**;**
Studies focused primarily on schizophrenia or severe psychiatric disorders;
Studies focused on animals.

Concept

Include:

*P*sychosocial barriers related to walking behavior and use of digital walking interventions;
Barriers including but not limited to motivation, self-efficacy, beliefs, attitudes, stigma, stereotypes, perceived norms, or social norms.
Barriers reported explicitly or implicitly (e.g. through qualitative findings or process evaluations).

Exclude:

Exclusive focus on physiological, biomechanical, or clinical outcomes;
Technical system performance without psychosocial data;
Studies addressing only facilitators with no reference to barriers;
Studies where psychosocial factors are mentioned only theoretically, with no empirical data.

Context

Include:

Digital interventions, such as mobile applications, wearable or pedometer-based technologies, web-based platforms, SMS- or text-based programs etc., that target walking/steps count.
Interventions delivered in community settings, home-based settings, outpatient or primary care settings.
All geographic locations and cultural contexts.

Exclude:

Non-digital or purely face-to-face interventions or multi-component interventions where walking is not primary objective/central;

Laboratory-based studies with no real-world or applied context; interventions implemented exclusively in inpatient or institutionalized settings (e.g., long-term residential care) unless digital and walking-focused;
Digital interventions targeting physical activity broadly without a walking or step-based component;
Interventions delivered exclusively in acute inpatient settings;
Tele-monitoring only systems.

Additional tips for screening process
Consistent with JBI guidance for scoping reviews, eligibility was assessed in several stages (title, abstract and full text). A sensitive approach was adopted during title and abstract screening to avoid premature exclusion, with full confirmation of PCC elements undertaken at the full-text stage.

Title & abstract screening tips

I. **Title screening** is conducted with the objective of excluding *only citations that are clearly irrelevant to the review question, such as protocols.* When relevance cannot be confidently determined based on the title alone, records will be retained and marked as “Maybe” for subsequent abstract screening.
II. **Abstract screening** is conducted to assess eligibility against the predefined inclusion and exclusion criteria. *Records are excluded when abstracts clearly indicate non-eligibility (PCC criteria).* In cases where eligibility remains uncertain or information is insufficient, studies are retained and advanced to full-text screening.

Population
If population unclear look for generic terms (eg., “patients”) with no age specified; chronic condition implied but not explicitly stated; mixed samples with chronic conditions with no clear stratification. Recruitment in a hospital/clinic setting.
Examples: "A digital walking intervention in primary care”; “Step-count interventions using smartphones”; "Patient engagement with..." etc.

<u>*Considered chronic with boundaries:*</u>

A) *Cancer patients* - considered chronic when it is conceptualized as a long-term condition affecting daily functioning, self-management, or behavior. Cancer patients, receiving immunotherapy – can be considered as chronic if no acute episode/end of life state present.
But cancer patients that receive chemotherapy are considered acute.
B) *Obesity* - considered chronic when: a) a chronic condition (explicitly or implicitly); b) the primary health condition of the sample; c) linked to long-term behavior change or daily activity.
Example: “Wearable-based physical activity promotion in people living with obesity”
Should be excluded if: a) framed as primary prevention in healthy populations; b) short-term weight loss programs.

Example: “High-intensity exercise for rapid weight reduction in young adults”

C) *COVID-19*: post-COVID conditions are considered chronic; Exclude studies during COVID pandemic.

D) *Parkinson’s disease* – is accepted as chronic irrespective of disease stage or duration, provided that Parkinson’s disease is identified as a long-term, non-communicable neurological condition.

E) *Stroke* – considered chronic if: a) ≥3–6 months after the event; b) long-term functional limitations are present; c) the focus is on long-term management rather than acute treatment.

F) Patients receiving *dialysis* are considered chronic as long as it is a maintenance therapy, long-term structured routine.

G) *HIV*- considered chronic, exclude only if the sample refers exclusively to acute.

H) *Psychiatric condition* – include only if it is a psychiatric comorbidity that exists within a chronic illness;

<u>*Exclude:*</u>

A) *Addition-related* conditions are excluded.

B) *End of life patients/care* (eg., hospice, palliative care) are excluded.

Concept

If concept is unclear: look for barriers reported implicitly (e.g. low adherence, poor engagement explained qualitatively); psychosocial issues discussed without being explicitly labelled as “barriers”; studies on acceptance/engagement where psychosocial dimensions are mentioned but not central.

Examples: "Adoption of mobile health technologies to promote walking..."; "“Implementation of..."; "Feasibility of..."; "“Behaviour change outcomes..."; "Experience and perspectives..." etc.

Context

If context is unclear: digital physical activity interventions where the walking component is not explicit but plausible; *multi-component interventions* if digital walking is a clearly identifiable component;

Examples: “Mobile health + to increase physical activity...”; “Effects of a smartphone-based physical activity program..”; “Physical activity promotion using wearable...”; “Technology-supported lifestyle program...”; “A home-based physical activity intervention...”; “A remotely delivered...”; “Step count changes following a physical activity...”; “Goal-setting or Feedback-based... to increase physical activity...” etc.

<u>*Exclude:*</u>

– Purely physiological, biomechanical, or pharmacological focus;
– Gait training; Resistance training; High-intensity training etc.

– If wearable device only for data collection.

Examples: “Digital resistance training intervention for older adults”; “High-intensity interval training delivered via mobile app”; “Physiotherapist-led walking sessions in rehabilitation clinics”; “Group-based exercise training delivered in community centers” etc.

Cleary unrelated topics

Exclude:

– Laboratory-only studies;

– Telemonitoring only (e.g. “remote monitoring of vital signs”);

Examples: “Biomechanical analysis of gait parameters using motion capture systems”; “Energy expenditure during treadmill walking: a laboratory study” etc.

Additional tips for full text screening

Objective of the full text screening is to confirm full eligibility against PCC.

Chronic adult population:

Adults ≥18 years OR explicitly older adults ≥65)

AND

Diagnosed chronic condition (single or multimorbidity): explicit medical diagnosis OR **r**ecruitment from chronic care pathway (e.g., cardiac rehab, COPD clinic, diabetes outpatient service).

Exclude: acute-only samples; healthy populations; mixed samples where chronic subgroup data cannot be disaggregated

Decision rule: Chronicity must be structurally embedded in the sample, not incidental.

Digital intervention:

Intervention must include a digital behaviour change component, such as mobile apps, wearables, web-based platforms, telehealth / telerehabilitation systems, automated messaging / digital coaching.

Exclude: purely face-to-face, paper-based, or non-digital programs.

Decision rule: Digital system drives the intervention logic.

Walking:

Walking/step count is primary outcome OR core behavioural target within intervention;

Walking is considered central when: step goals drive the intervention; engagement metrics are based on walking; intervention design revolves around increasing walking behaviour;

*Exclude: w*alking is one minor component of general lifestyle change, physical activity broadly targeted without walking-specific emphasis; walking only measured descriptively.

Decision rule: If walking is removed, the intervention loses its conceptual logic.

Psychosocial variable:

A psychosocial variable must: be measured (quantitatively or qualitatively), be linked to walking behaviour and/or digital engagement, function as a barrier (explicitly or conceptually).

Decision rule: Psychosocial barrier must explain friction in behavioural adoption or maintenance.

SECTION 3. Data extraction tool

Mixed population as described
Copy past from the text.

If results for the chronic sample differentiated from the mixed population?

- ◯ YES
- ◯ NO
- ◯ N/A
- ◯ Other

Study context
Copy from the text;
Example: community / home-based / outpatient / primary care / mixed.

Digital intervention type/context
Example: mobile app / wearable / pedometer / web-based / SMS / mixed.

What components are delivered digitally?

What Behaviour Change Techniques were used within digital intervention/context?

Intervention duration
N/A if no intervention; N/D if no data reported or reported elsewhere.

Comparator (if present)

Level of digital support

- ☐ Fully automated
- ☐ Blended
- ☐ Clinician-supported
- ☐ Other

Verbatim quote regarding level of digital support.

Primary outcome of the study

Secondary outcome of the study

Walking focus of intervention

- ◯ Primary focus
- ◯ Secondary focus
- ◯ Broad PA focus with walking
- ◯ Unclear

Walking-related target behaviour
The specific walking activity (mobility action) that the intervention, assessment, or study is aiming to change, support, improve, monitor, or understand.

Primary walking-related outcome
The main walking- or mobility-related outcome that the study evaluates to assess the effect of the intervention or to answer the main research question.

Walking measurement tool

Other PA reported (not walking)

Mixed population as described
Copy past from the text.

If results for the chronic sample differentiated from the mixed population?

- ○ YES
- ○ NO
- ○ N/A
- ○ Other

Study context
Copy from the text;
Example: community / home-based / outpatient / primary care / mixed.

Digital intervention type/context
Example: mobile app / wearable / pedometer / web-based / SMS / mixed.

What components are delivered digitally?

What Behaviour Change Techniques were used within digital intervention/context?

Intervention duration
N/A if no intervention; N/D if no data reported or reported elsewhere.

Comparator (if present)

Level of digital support

- ☐ Fully automated
- ☐ Blended
- ☐ Clinician-supported
- ☐ Other

Verbatim quote regarding level of digital support.

Primary outcome of the study

Secondary outcome of the study

Walking focus of intervention

- ○ Primary focus
- ○ Secondary focus
- ○ Broad PA focus with walking
- ○ Unclear

Walking-related target behaviour
The specific walking activity (mobility action) that the intervention, assessment, or study is aiming to change, support, improve, monitor, or understand.

Primary walking-related outcome
The main walking- or mobility-related outcome that the study evaluates to assess the effect of the intervention or to answer the main research question.

Walking measurement tool

Other PA reported (not walking)

Psychosocial barrier identified
Report barrier as stated in the text;

RW barrier label coding

RW barrier domain coding

Barrier definition as provided by authors
Copy past from the text.
N/D is not provided;

Barrier conceptual framing by author
Eg., determinant, obstacle

Barrier measurement method
Qualitative theme / Validated scale / Ad hoc item / Other

Barrier measurement tool/item/question (if provided)
N/D if not stated.

Barrier assessed at what T?
Baseline / during intervention / post-intervention / only discussed.

Barrier linked to outcomes
Copy past as reported in the text;
N/D if not mentioned.

How linked to barrier outcome / process measured

Direction of association (description of relation) with an outcome
Negative / Positive / Mixed / Unclear;
Copy past as stated in the text;

Statistical / qualitative evidence for barrier
As reported in the text;

Summary of the results related to the barrier

SECTION 4.

Table S6. Intervention Characteristics of Included Studies (N = 28)

Intervention Characteristics[1]	Category	N (%)
**Intervention type**	Digital interventions with human support	11 (39.3)
	Web-based interventions combined with pedometers	8 (28.6)
	App–wearable combinations	6 (21.4)
	Mobile applications alone	3 (10.7)
**Level of digital support**	Clinician/human-supported	13 (46.4)
	Fully automated	13 (46.4)
	N/A	2 (7.1)
**Walking focus**	Primary focus	18 (64.3)
	Walking within broader physical activity focus	10 (35.7)
**Walking-related target behaviour or goal**	Increase walking	21 (75.0)
	Maintain or increase walking	4 (14.3)
	Increase walking and other physical activity	3 (10.7)
**Walking measurement**	Consumer wearable activity tracker	9 (32.1)
	Mixed measurement tools	8 (28.6)
	Pedometer	6 (21.4)
	Accelerometer / research-grade activity monitor	1 (3.6)
	Smartphone-based activity tracking	1 (3.6)

	Self-reported physical activity questionnaire	1 (3.6)
	N/A	1 (3.6)
	N/R	1 (3.6)
**Study context**	Hybrid	10 (35.7)
	Outpatient / ambulatory care setting	10 (35.7)
	Community / home-based setting	8 (28.6)

Note. N/A = Not applicable; N/R = Not reported. (1) Intervention characteristics were coded by the reviewers for data presentation; details of coding and grouping are provided in the Methods section.

Table S7. Reviewer-Defined Grouped Behaviour Change Technique (BCT) Categories

BCT[1] group category	Group description	Typical intervention elements included	Theoretical rational[2]	Example studies[3]
**Goal setting and planning**	Techniques that define behavioural targets and support planning for behaviour enactment.	Goal setting, step goals, behavioural goal setting, action planning, review of goals, proximal goals, graded goals when used as structured progression.	Informed by BCT Taxonomy v1 and self-regulation and control theories. These techniques establish the reference standard or goal against which behaviour can be monitored and adjusted.	Robinson et al., 2024; Höchsmann et al., 2019; Kaye et al., 2014; Bartlett et al., 2017
**Self-monitoring and feedback**	Techniques that support tracking, observation, and evaluation of behaviour or outcomes over time.	Self-monitoring of behaviour, self-monitoring of outcomes, feedback on behaviour, feedback on progress, automated feedback, iterative feedback, real-time biofeedback.	Grounded in self-regulation and control theory, in which behaviour change is supported through monitoring performance. Also aligned with BCT Taxonomy v1 self-monitoring and feedback techniques.	Golbus et al., 2026; Robinson et al., 2024; Nguyen et al., 2017; Vorrink et al., 2016; Wulfovich et al., 2019
**Prompts and reminders**	Techniques that cue action, maintain awareness of the target behaviour, or prompt re-engagement.	Prompts and cues, reminders, nudges, SMS reminders, email notifications, app notifications, prompt intention formation, prompt review of behaviour when used as cues.	Supported by BCT Taxonomy v1 and self-regulation and control theories. These techniques help maintain goal salience and support enactment when attention or motivation may lapse.	Golbus et al., 2026; Syrjälä et al., 2021; Tincopa et al., 2024; Bartlett et al., 2017; Wulfovich et al., 2019
**Tailoring and personalization**	Techniques that adapt goals, feedback, progression, or content to individual needs, performance, or context.	Personalised goals, tailored feedback, tailored progression, personalised educational content, personalised goal adjustment, context-aware content.	A reviewer-defined synthesis category, informed by BCT Taxonomy v1 and by self-regulation logic. Tailoring increases the fit between intervention demands and individual circumstances, thereby strengthening behavioural regulation and intervention relevance.	Robinson et al., 2020; Höchsmann et al., 2019; Syrjälä et al., 2021; Roberts et al., 2019; Vorrink et al., 2016
**Education and instruction**	Techniques that provide knowledge, explanation, or procedural guidance.	Educational tips, instruction on how to perform behaviour, information about health consequences, self-management information, demonstration of behaviour.	Mainly supported by BCT Taxonomy v1 instruction- and knowledge-related techniques. These components aim to increase understanding of the behaviour and how it can be performed.	Robinson et al., 2024; Robinson et al., 2020; Golbus et al., 2026; Roberts et al., 2019; Bartlett et al., 2017
**Motivational support and reinforcement**	Techniques intended to enhance willingness, persistence, effort, or positive orientation toward behaviour change.	Motivational messages, encouragement, positive reinforcement, positive feedback, rewards, incentives, persuasive messages.	Informed by BCT Taxonomy v1 reinforcement-related techniques and by broader motivational perspectives, including Social Cognitive Theory and Self-Determination Theory, where reinforcement and supportive messaging may strengthen engagement and persistence.	Robinson et al., 2024; Robinson et al., 2020; Kaye et al., 2014; Lambert et al., 2024; Okwose et al., 2020
**Social support**	Techniques that use interpersonal encouragement, peer connection, accountability,	Peer support, online community forum, social connectedness, support from others, accountability, collaboration, encouragement from peers or groups.	Supported by BCT Taxonomy v1 social support techniques and by Self-Determination Theory, especially relatedness, as well as broader social-behavioural perspectives that emphasise the supportive role of interpersonal context.	Robinson et al., 2024; Robinson et al., 2020; Roberts et al., 2019; Lambert et al., 2024; Okwose et al., 2020

	or relatedness to support behaviour change.			
**Coaching and human support**	Human-delivered behavioural support embedded within a digital intervention.	Clinician support, troubleshooting, health coaching, regular support contact.	A reviewer-defined synthesis category. It was retained because some interventions combined digital delivery with direct human input that supported goal pursuit, accountability, problem solving, or self-regulation.	Höchsmann et al., 2019; Syrjälä et al., 2021; Kaye et al., 2014; Nguyen et al., 2017
**Problem solving and barrier management**	Techniques that support identification of barriers, management of setbacks, and generation of strategies to sustain behaviour.	Problem solving, discussion of barriers and facilitators, strategies to become more active, dealing with setbacks.	Supported by BCT Taxonomy v1 problem solving techniques and by self-regulation approaches, in which behavioural adjustment is required when obstacles interfere with progress.	Roberts et al., 2019; Kaye et al., 2014; Frensham et al., 2018; Nguyen et al., 2017; Okwose et al., 2020
**Self-regulation and self-efficacy support**	Techniques intended to strengthen confidence, perceived capability, autonomy, competence, or self-regulatory capacity.	Self-efficacy discussion, choice and control support, mastery support, modelling, social persuasion, competence support, autonomy-supportive framing.	A reviewer-defined synthesis category supported by Social Cognitive Theory for self-efficacy-related elements and by Self-Determination Theory for autonomy and competence support. Also compatible with self-regulation perspectives where behavioural persistence depends on regulatory capacity.	Robinson et al., 2019; Roberts et al., 2019; Bamonti et al., 2023; Okwose et al., 2020
**Gamification and incentives**	Techniques that use game-like mechanics, symbolic rewards, competition, or incentive structures to increase engagement.	Points, levels, badges, awards, competitions, gamification, nonspecific rewards, nonspecific incentives, staged competitions.	Anchored partly in BCT Taxonomy v1 reward and incentive techniques but retained as a separate synthesis category because digital walking interventions often implemented these features as a distinct engagement layer.	Tincopa et al., 2024; Bamonti et al., 2023; Lambert et al., 2024

Note. (1) BCT - Behaviour Change Technique; (2) Theoretical rational - grouped BCT categories were reviewer-defined synthesis categories informed by the Behaviour Change Technique Taxonomy v1 [1], Self-Determination Theory [2], Social Cognitive Theory [3] by self-regulation and control-theory perspectives [4]. Evidence citations are illustrative examples from included studies and are not intended to represent an exhaustive mapping of all studies contributing to each grouped category. Some categories, particularly coaching and human support, tailoring and personalization, and self-regulation and self-efficacy support, reflect synthesis-level functional groupings rather than single discrete BCT Taxonomy v1 classes. (3) Example studies inlcude selected studies, not all studies that reported each BCT group category.

References

1. Michie, S., Richardson, M., Johnston, M., Abraham, C., Francis, J., Hardeman, W., Eccles, M. P., Cane, J., & Wood, C. E. (2013). The behavior change technique taxonomy (v1) of 93 hierarchically clustered techniques: building an international consensus for the reporting

of behavior change interventions. Annals of behavioral medicine: a publication of the Society of Behavioral Medicine, 46(1), 81–95. https://doi.org/10.1007/s12160-013-9486-6

2. Ryan, R. M., & Deci, E. L. (2000). Self-determination theory and the facilitation of intrinsic motivation, social development, and well-being. The American psychologist, 55(1), 68–78. https://doi.org/10.1037//0003-066x.55.1.68
3. Bandura, A., & National Inst of Mental Health. (1986). Social foundations of thought and action: A social cognitive theory. Prentice-Hall, Inc.
4. Carver, C. S., & Scheier, M. F. (1982). Control theory: a useful conceptual framework for personality-social, clinical, and health psychology. Psychological bulletin, 92(1), 111–135.

Table S8. Behavioural Change Techniques Group Category Reported in the Included Studies (N = 28)

BCT[1] domain	Studies using technique[2] n (%)	Level of digital support n (%)		
		**Clinician/human-supported**	**Fully automated**	**N/A**
Self-monitoring and feedback	28 (100.0)	13 (46.4)	13 (46.4)	2 (7.1)
Goal setting and planning	25 (89.3)	12 (42.9)	12 (42.9)	1 (3.6)
Motivational support and reinforcement	17 (60.7)	6 (21.4)	10 (35.7)	1 (3.6)
Social support	15 (53.6)	4 (14.3)	10 (35.7)	1 (3.6)
Tailoring and personalization	12 (42.9)	6 (21.4)	5 (17.9)	1 (3.6)
Prompts and reminders	11 (39.3)	5 (17.9)	5 (17.9)	1 (3.6)
Education and instruction	10 (35.7)	2 (7.1)	7 (25.0)	1 (3.6)
Problem solving and barrier management	6 (21.4)	5 (17.9)	1 (3.6)	0 (0.0)
Coaching and human support	5 (17.9)	4 (14.3)	1 (3.6)	0 (0.0)
Self-regulation and self-efficacy support	4 (14.3)	3 (10.7)	1 (3.6)	0 (0.0)
Gamification and incentives	4 (14.3)	1 (3.6)	3 (10.7)	0 (0.0)

Note. (1) BCT – Behavioural Change Techniques; (2) - n and % are calculated from total n=28 without N/A.

Table S9. Intervention BCT Techniques and Components Across Levels of Digital Support and Walking Target Behaviours (N=28)

BCT domain	Walking-related target goal	Level of digital support	N (%)	Total[1] N (%)
**Self-monitoring and feedback**	Increase walking	Clinician/human-supported	9 (32.1)	28 (100.0)
		Fully automated	10 (35.7)	
		N/A	2 (7.1)	
	Maintain or increase walking	Clinician/human-supported	3 (10.7)	
		Fully automated	1 (3.6)	
		N/A	0 (0.0)	
	Increase walking and other PA	Clinician/human-supported	1 (3.6)	
		Fully automated	2 (7.1)	
		N/A	0 (0.0)	
**Goal setting and planning**	Increase walking	Clinician/human-supported	9 (32.1)	25 (89.3)
		Fully automated	9 (32.1)	
		N/A	1 (3.6)	
	Maintain or increase walking	Clinician/human-supported	2 (7.1)	
		Fully automated	1 (3.6)	
		N/A	0 (0.0)	
	Increase walking and other PA	Clinician/human-supported	1 (3.6)	
		Fully automated	2 (7.1)	
		N/A	0 (0.0)	
**Prompts and reminders**	Increase walking	Clinician/human-supported	2 (7.1)	11 (39.3)
		Fully automated	3 (10.7)	
		N/A	1 (3.6)	
	Maintain or increase walking	Clinician/human-supported	2 (7.1)	
		Fully automated	0 (0.0)	
		N/A	0 (0.0)	
	Increase walking and other PA	Clinician/human-supported	1 (3.6)	
		Fully automated	2 (7.1)	
		N/A	0 (0.0)	
**Tailoring and personalization**	Increase walking	Clinician/human-supported	3 (10.7)	12 (42.9)

		Fully automated	4 (14.3)	
		N/A	1 (3.6)	
	Maintain or increase walking	Clinician/human-supported	2 (7.1)	
		Fully automated	0 (0.0)	
		N/A	0 (0.0)	
	Increase walking and other PA	Clinician/human-supported	1 (3.6)	
		Fully automated	1 (3.6)	
		N/A	0 (0.0)	
**Education and instruction**	Increase walking	Clinician/human-supported	2 (7.1)	10 (35.7)
		Fully automated	5 (17.9)	
		N/A	1 (3.6)	
	Maintain or increase walking	Clinician/human-supported	0 (0.0)	
		Fully automated	1 (3.6)	
		N/A	0 (0.0)	
	Increase walking and other PA	Clinician/human-supported	0 (0.0)	
		Fully automated	1 (3.6)	
		N/A	0 (0.0)	
**Motivational support and reinforcement**	Increase walking	Clinician/human-supported	5 (17.9)	17 (60.7)
		Fully automated	7 (25.0)	
		N/A	1 (3.6)	
	Maintain or increase walking	Clinician/human-supported	1 (3.6)	
		Fully automated	1 (3.6)	
		N/A	0 (0.0)	
	Increase walking and other PA	Clinician/human-supported	0 (0.0)	
		Fully automated	2 (7.1)	
		N/A	0 (0.0)	
**Social support**	Increase walking	Clinician/human-supported	4 (14.3)	15 (53.6)
		Fully automated	7 (25.0)	
		N/A	1 (3.6)	
	Maintain or increase walking	Clinician/human-supported	0 (0.0)	

		Fully automated	1 (3.6)	
		N/A	0 (0.0)	
	Increase walking and other PA	Clinician/human-supported	0 (0.0)	
		Fully automated	2 (7.1)	
		N/A	0 (0.0)	
**Coaching and human support**	Increase walking	Clinician/human-supported	3 (10.7)	5 (17.9)
		Fully automated	1 (3.6)	
		N/A	0 (0.0)	
	Maintain or increase walking	Clinician/human-supported	0 (0.0)	
		Fully automated	0 (0.0)	
		N/A	0 (0.0)	
	Increase walking and other PA	Clinician/human-supported	1 (3.6)	
		Fully automated	0 (0.0)	
		N/A	0 (0.0)	
**Problem solving and barrier management**	Increase walking	Clinician/human-supported	5 (17.9)	6 (21.4)
		Fully automated	1 (3.6)	
		N/A	0 (0.0)	
**Self-regulation and self-efficacy support**	Increase walking	Clinician/human-supported	3 (10.7)	4 (14.3)
		Fully automated	1 (3.6)	
		N/A	0 (0.0)	
**Gamification and incentives**	Increase walking	Clinician/human-supported	1 (3.6)	4 (14.3)
		Fully automated	2 (7.1)	
	Increase walking and other PA	Fully automated	1 (3.6)	

Note. (1) Total N (%) is reported per BCT group reported in included studies (n=28); BCT – Behaviour change technique; PA – Physical activity; N/A – Not applicable.

Table S10. Barrier Conceptualization and Identification (N = 105)

Category[1]	Subcategory	N (%)
Barrier identification type[2]	Explicit	59 (56.2)
	Implicit	46 (43.8)
Barrier assessment method	Qualitative interview / focus-group theme	70 (66.7)
	Validated scale	12 (11.4)
	Open-ended participant feedback / free-text response	7 (6.7)
	Mixed-methods integrated finding	5 (4.8)
	Study-specific self-report item(s)	5 (4.8)
	Other / narrative/ contextual discussion	6 (5.7)

Note. (1) Categorisation procedures are described in the Methods section; (2) Barriers were classified as explicit when directly labelled by the study authors as barriers or related terms (e.g., obstacle, constraint, challenge), and as implicit when not explicitly named as barriers but shown/discussed to hinder behaviour or intervention-related processes.

Table S11. Reviewer-Generated Barrier Domains Mapped to COM-B and TDF, With Illustrative Coded Examples (N=105)

Higher-order barrier domain	Barrier instances, n (%)	Dominant COM-B assignment	Dominant TDF assignment	Coding interpretation used in synthesis	Illustrative coded examples from included studies
**Belief-based barriers**	22 (21.0)	Motivation (17/22), with minor coding to Capability and Opportunity	Primarily Beliefs about Consequences (15/22), followed by Knowledge and Beliefs about Capabilities	Used when the barrier primarily reflected negative cognitive appraisals about walking or the intervention, including doubts about usefulness, credibility, value, privacy, or one's own walking capability	*Perceived lack of value of trackers* and skepticism toward trackers (Gualtieri et al., 2016); *negative beliefs/catastrophizing about walking ability* (Bamonti et al., 2025); *intervention perceived as too simplistic/low credibility* (Lambert et al., 2024)
**Emotional barriers**	22 (21.0)	Motivation (21/22)	Primarily Emotion (21/22)	Used when the dominant mechanism was affective, such as fear, frustration, shame, guilt, stress, or aversion, directly interfering with behavioural initiation, persistence, or intervention engagement	*Health-related fear of physical activity* (Golbus et al., 2026); *frustration with tracker feedback limitations* (Gualtieri et al., 2016); *stress and uncomfortableness from pedometer monitoring* (Marklund et al., 2021); *Anxiety related to shortness of breath and physical activity* (Bamonti et al., 2025)
**Self-regulation and goal-management barriers**	16 (15.2)	Motivation (13/16), with minor coding to Capability and Opportunity	Mainly Goals (4/16) and Beliefs about Capabilities (4/16), with additional coding to Reinforcement, Intentions, and Behavioural Regulation	Used when the barrier reflected difficulty setting, calibrating, monitoring, or persisting with behavioural goals, especially where targets felt unrealistic or confidence in goal attainment was low	*Step goals perceived as too high* (Robinson et al., 2020); *not feeling comfortable reaching step-count goals* (Robinson et al., 2020); *reduced motivation from auto-adjusted step goals* (Nguyen et al., 2017); *avoiding disappointment in not achieving goals* (Bentley et al., 2020)
**Social and relational barriers**	12 (11.4)	Opportunity (9/12), with some coding to Motivation	Primarily Social Influences (9/12)	Used when the dominant mechanism was interpersonal or relational, including lack of encouragement, fear of judgment, negative peer dynamics, or insufficient ongoing support	*Need for ongoing support* (Gualtieri et al., 2016); *need for peer/social support* (Ginossar et al., 2021); *fear of judgment by others* (Wulfovich et al., 2019); *social isolation* (Frensham et al., 2018)

**Digital capability and usability barriers**	11 (10.5)	Capability (8/11), with some coding to Motivation	Split mainly between Skills (4/11) and Beliefs about Capabilities (4/11)	Used when the barrier primarily concerned practical ability, confidence, or literacy needed to operate the digital tool as intended	*Difficulties using the pedometer* (Robinson et al., 2020); *limited digital literacy and low confidence using technology* (Ginossar et al., 2021); *limited technical abilities/low technology self-confidence* (Nguyen et al., 2017)
**Motivation-related barriers**	11 (10.5)	Motivation (11/11)	Primarily Intentions (6/11), with some Behavioural Regulation and Beliefs about Consequences	Used when the barrier reflected reduced drive, weak commitment, amotivation, waning enthusiasm, or declining willingness to sustain intervention use or walking goals	*Amotivation* and *controlled motivation* (Golbus et al., 2026); *low motivation* (Marklund et al., 2021; Bamonti et al., 2025); *waning enthusiasm for pedometer use* (Lambert et al., 2024)
**Support and contextual resource barriers**	11 (10.5)	Opportunity (6/11), with some coding to Motivation and Capability	Mainly Social Influences (5/11) and Environmental Context and Resources (3/11)	Used when barriers arose from external conditions surrounding intervention uptake or maintenance, such as lack of time, caregiver/professional support, monitoring burden, or low trust in device outputs	*Time constraints* (Robinson et al., 2020); *lack of caregiver involvement* (Khairat et al., 2026); *insufficient support from physiotherapists* (Vorrink et al., 2016); *lack of trust in the readings* (Doyle et al., 2021)

Note. Frequencies are based on the 105 verbatim psychosocial barrier instances extracted from the included studies.

Table S12. Barrier domains by study type (N = 105)

High-domain barrier group	Experimental study n (%)	Pilot and feasibility study n (%)	Observational study n (%)	Qualitative study n (%)	Mixed-method study n (%)	Secondary analysis n (%)	Total n (%)
Belief-based barriers	1 (1.0)	1 (1.0)	1 (1.0)	12 (11.4)	7 (6.7)	0 (0.0)	22 (21.0)
Digital capability and usability barriers	2 (1.9)	2 (1.9)	0 (0.0)	5 (4.8)	1 (1.0)	1 (1.0)	11 (10.5)
Emotional barriers	2 (1.9)	4 (3.8)	1 (1.0)	9 (8.6)	4 (3.8)	2 (1.9)	22 (21.0)
Motivation-related barriers	1 (1.0)	1 (1.0)	2 (1.9)	4 (3.8)	2 (1.9)	1 (1.0)	11 (10.5)
Self-regulation and goal-management barriers	3 (2.9)	3 (2.9)	0 (0.0)	4 (3.8)	4 (3.8)	2 (1.9)	16 (15.2)
Social and relational barriers	0 (0.0)	1 (1.0)	0 (0.0)	7 (6.7)	4 (3.8)	0 (0.0)	12 (11.4)
Support and contextual resource barriers	1 (1.0)	3 (2.9)	0 (0.0)	3 (2.9)	3 (2.9)	1 (1.0)	11 (10.5)
Total n (%)	10 (9.5)	15 (14.2)	4 (3.8)	44 (41.9)	25 (23.8)	7 (6.7)	

Note. Study grouping is described in the Methods section.

Table S13. Barrier Domains and Related Assessment Methods

**Psychosocial barrier domain**	**Qualitative interview / focus-group theme n (%)**	**Open-ended feedback n (%)**	**Validated scale n (%)**	**Study-specific item n (%)**	**Mixed methods integrated n (%)**	**Other / narrative / contextual n (%)**	**Total n (%)**
Belief-based barriers	17 (16.2)	1 (1.0)	3 (2.9)	0 (0.0)	1 (1.0)	0 (0.0)	22 (21.0)
Digital capability and usability	7 (6.7)	1 (1.0)	0 (0.0)	1 (1.0)	0 (0.0)	2 (1.9)	11 (10.5)
Emotional barriers	16 (15.2)	0 (0.0)	4 (3.8)	1 (1.0)	0 (0.0)	1 (1.0)	22 (21.0)
Motivation-related barriers	5 (4.8)	2 (1.9)	2 (1.9)	0 (0.0)	1 (1.0)	1 (1.0)	11 (10.5)
Self-regulation and goal-management	8 (7.6)	0 (0.0)	3 (2.9)	2 (1.9)	3 (2.9)	0 (0.0)	16 (15.2)
Social and relational barriers	9 (8.6)	3 (2.9)	0 (0.0)	0 (0.0)	0 (0.0)	0 (0.0)	12 (11.4)
Support and contextual resources	8 (7.6)	0 (0.0)	0 (0.0)	1 (1.0)	0 (0.0)	2 (1.9)	11 (10.5)

Note. The categorisation and grouping of assessment methods are described in detail in the Methods section.

Table S14. Barriers by Related to Them Outcomes (N = 105)

Barrier-related outcomes	N of instances	% of Total	Examples
Walking and PA outcome	30	28.6	Lower daily step count, 12-hour step count, lower physical activity participation;
Engagement and use	25	23.8	Early intervention engagement, perceived ease of finding time for website use, willingness to try tracker;
Adherence, retention and participation	18	17.1	Goal attainment difficulty, reduced study retention, withdrawal;
Acceptability, usability and fit	14	13.3	Perceiving goals as too high, technology acceptance and use, positive attitude toward technology;
Psychological and emotional response	11	10.5	Goal-related confidence and comfort, goal adherence driven by shame avoidance;
Broader recovery, HRQOL and self-management	4	3.8	Need for peer support, broader recovery support needs, broader recovery and well-being support;
Not specified	3	2.9	-

Note. Not specified includes N/A (not applicable), N/D (not described), and N/R (not reported).

Table S15. Identified Barrier Domains by Outcome (N=105)

High-domain barrier group	Intervention-process outcomes n (%)	Outcome-related %	Behavioural outcomes n (%)	Outcome-related %	Psychological and broader self-management outcomes n (%)	Outcome-related %	Not specified[1] n (%)	Total within barrier n (%)
Belief-based barriers	13 (59.1)	22.8	6 (27.3)	20.0	3 (13.6)	20.0	0 (0.0)	22 (21.0)
Digital capability and usability barriers	9 (81.8)	15.8	0 (0.0)	0.0	1 (9.1)	6.7	1 (9.1)	11 (10.5)
Emotional barriers	9 (40.9)	15.8	9 (40.9)	30.0	3 (13.6)	20.0	1 (4.5)	22 (21.0)
Motivation-related barriers	5 (45.5)	8.8	4 (36.4)	13.3	1 (9.1)	6.7	1 (9.1)	11 (10.5)
Self-regulation and goal-management barriers	7 (43.8)	12.3	4 (25.0)	13.3	5 (31.3)	33.3	0 (0.0)	16 (15.2)
Social and relational barriers	6 (50.0)	10.5	4 (33.3)	13.3	2 (16.7)	13.3	0 (0.0)	12 (11.4)
Support and contextual resource barriers	8 (72.7)	14.0	3 (27.3)	10.0	0 (0.0)	0.0	0 (0.0)	11 (10.5)
Total n (%)	57 (54.3)	-	30 (28.6)	-	15 (14.3)	-	3 (2.9)	105 (100.0)

Note. Percentages are calculated within each barrier domain; Total percentages are calculated by outcome based on the 105 identified barrier instances. (1) Not specified includes N/A (not applicable), N/D (not described), and N/R (not reported).

Table S16. Psychosocial Barrier Domains by Two Levels of Outcome-Related Categories With Illustrative Examples (N = 105)

Barrier domain	Barrier-related outcome group	N (%)	Barrier-related outcomes within groups	N (%)	Examples of extracted instances
**Belief-based barriers**	Behavioural outcomes	6 (27.3)	Walking/PA outcome	6 (100.0)	Physical activity barriers, lack of awareness of physical limitations;
	Intervention-process outcomes	13 (59.1)	Engagement/use	6 (46.2)	Perceived lack of value of trackers, the Fitbit itself was not motivational;
			Acceptability/usability/fit	5 (38.5)	Scepticism toward trackers, the novelty of the Fitbit wore off;
			Adherence/retention/participation	2 (15.4)	Negative beliefs about the intervention, concerns about device relevance;
	Psychological and broader self-management outcomes	3 (13.6)	Psychological/emotional response	1 (33.3)	Feeling unmotivated when activity was not captured;
			Broader recovery/HRQOL/self-management	2 (66.7)	More information did not always improve motivation, HealthScore metrics are not comprehensive;
	Not specified	0 (0.0)	Not specified	0 (0.0)	-
**Digital capability and usability barriers**	Behavioural outcomes	0 (0.0)	Walking/PA outcome	0 (0.0)	-
	Intervention-process outcomes	9 (81.8)	Engagement/use	3 (33.3)	Difficulties using the pedometer, trouble using the smartphone or app;
			Acceptability/usability/fit	3 (33.3)	Difficulty using technology, frustration with device/app complexity;
			Adherence/retention/participation	3 (33.3)	Limited digital and self-management skills;
	Psychological and broader self-management outcomes	1 (9.1)	Psychological/emotional response	0 (0.0)	-
			Broader recovery/HRQOL/self-management	1 (100.0)	Difficulty integrating technology into daily routines;

	Not specified	1 (9.1)	Not specified	1 (100.0)	-
**Emotional barriers**	Behavioural outcomes	9 (40.9)	Walking/PA outcome	9 (100.0)	Fear of physical activity, depression;
	Intervention-process outcomes	9 (40.9)	Engagement/use	5 (55.6)	Shame and guilt regarding smoking and COPD, intervention-related worry;
			Acceptability/usability/fit	1 (11.1)	Frustration with tracker feedback;
			Adherence/retention/participation	3 (33.3)	Fear of undertaking physical activity, depression affecting adherence;
	Psychological and broader self-management outcomes	3 (13.6)	Psychological/emotional response	3 (100.0)	Emotional challenges, feeling discouraged;
			Broader recovery/HRQOL/self-management	0 (0.0)	-
	Not specified[1]	1 (4.5)	Not specified	1 (100.0)	Stress and uncomfortableness from pedometer monitoring;
**Motivation-related barriers**	Behavioural outcomes	4 (36.4)	Walking/PA outcome	4 (100.0)	Amotivation; controlled motivation;
	Intervention-process outcomes	5 (45.5)	Engagement/use	3 (60.0)	Low motivation to use the app, reduced engagement with step-count graphs;
			Acceptability/usability/fit	0 (0.0)	
			Adherence/retention/participation	2 (40.0)	Lack of motivation to sustain participation; messages were impersonal and repetitive;
	Psychological and broader self-management outcomes	1 (9.1)	Psychological/emotional response	1 (100.0)	Low motivation following non-tailored support;
			Broader recovery/HRQOL/self-management	0 (0.0)	-
	Not specified	1 (9.1)	Not specified	1 (100.0)	
**Self-regulation and goal-management barriers**	Behavioural outcomes	4 (25.0)	Walking/PA outcome	4 (100.0)	Low self-efficacy; lack of confidence;

	Intervention-process outcomes	7 (43.8)	Engagement/use	1 (14.3)	Avoiding disappointment in not achieving goals;
			Acceptability/usability/fit	1 (14.3)	Step goals perceived as too high;
			Adherence/retention/participation	5 (71.4)	Frustration from competing priorities, demotivation from not meeting targets;
	Psychological and broader self-management outcomes	5 (31.3%)	Psychological/emotional response	5 (100.0)	Not feeling comfortable reaching step-count goals;
			Broader recovery/HRQOL/self-management	0 (0.0)	-
	Not specified	0 (0.0)	Not specified	0 (0.0)	-
**Social and relational barriers**	Behavioural outcomes	4 (33.3)	Walking/PA outcome	4 (100.0)	Technology cannot replace human interaction; negative attention from others;
	Intervention-process outcomes	6 (50.0)	Engagement/use	4 (66.7)	Need for ongoing support; difficulty maintaining social contact;
			Acceptability/usability/fit	2 (33.3)	Discouragement from teammate participation;
			Adherence/retention/participation	0 (0.0)	-
	Psychological and broader self-management outcomes	2 (16.7)	Psychological/emotional response	1 (50.0)	Need for peer/social support;
			Broader recovery/HRQOL/self-management	1 (50.0)	Lack of social integration/support;
	Not specified	0 (0.0)	Not specified	0 (0.0)	-
**Support and contextual resource barriers**	Behavioural outcomes	3 (27.3)	Walking/PA outcome	3 (100.0)	Lack of encouragement; technology-based adaptation challenges;
	Intervention-process outcomes	8 (72.7)	Engagement/use	3 (37.5)	Time constraints; many participants did not use the app;
			Acceptability/usability/fit	2 (25.0)	Choice confusion;
			Adherence/retention/participation	3 (37.5)	Lack of caregiver involvement; fear of losing therapist contact;
	Psychological and broader self-management outcomes	0 (0.0)	Psychological/emotional response	0 (0.0)	-

			Broader recovery/HRQOL/self-management	0 (0.0)	-
	Not specified	0 (0.0)	Not specified	0 (0.0)	-

Note. (1) Not specified in this case indicates instance where the influence was described as ambivalent by the study authors; Not specified includes N/A (not applicable), N/D (not described), and N/R (not reported).

Table S17. Independent Verification of Extracted Data (N = 6)

**Data-charting category verified**	**Fields included in verification**	**Item-level checks, n**	**Agreement n**	**Disagreement n**	**Agreement %**	**Summary**
Study characteristics	Study context / setting	6	6	0	100.0%	No discrepancies identified
Intervention characteristics	Intervention description; intervention effectiveness / operationalisation; walking target behaviour	18	16	2	88.9%	Discrepancies mainly concerned wording of intervention effectiveness and specification of step-count/walking-related targets
Behaviour change components (BCTs)	Behaviour change components or BCT grouping extracted from the intervention description	6	4	2	66.7%	Discrepancies mainly concerned whether specific elements should be classified as BCTs or as broader intervention/retention strategies
Psychosocial barrier identification	Barrier label / description; evidence source for barrier; specific item, question, or measure; assessment timing; supporting evidence / interpretation	76	75	1	98.7%	One discrepancy concerned clarification of the specific item or measure used to identify the barrier
Barrier–outcome & intervention-process linkage	Outcomes extracted; linkage between barrier and outcome or intervention process	38	36	2	94.7%	Discrepancies mainly concerned outcome categorisation
Total	All verified data-charting fields	144	137	7	95.1%	

Note: The random sample was generated in Microsoft Excel. This procedure was conducted as an independent verification of the extracted data against the original full-text articles. Percent agreement was calculated at the item level across the verified data-charting fields.

Table S18. Descriptive Evidence Distribution Across Psychosocial Barrier Domains by Contributing Studies (N = 28)

Category	Belief-based	Digital capability and usability	Emotional	Motivation-related	Self-regulation and goal-management	Social and relational	Support and contextual resource	Total N of studies
**Clinical populations**								
Cancer survivors	5 (17.9%)	3 (10.7%)	4 (14.3%)	1 (3.6%)	4 (14.3%)	4 (14.3%)	3 (10.7%)	9 (32.1%)
COPD	3 (10.7%)	4 (14.3%)	6 (21.4%)	5 (17.9%)	4 (14.3%)	1 (3.6%)	4 (14.3%)	11 (39.3%)
Heart failure	2 (7.1%)	0 (0.0%)	2 (7.1%)	1 (3.6%)	0 (0.0%)	1 (3.6%)	1 (3.6%)	2 (7.1%)
Type 2 diabetes	1 (3.6%)	0 (0.0%)	0 (0.0%)	1 (3.6%)	0 (0.0%)	1 (3.6%)	0 (0.0%)	1 (3.6%)
Multiple chronic conditions	4 (14.3%)	2 (7.1%)	3 (10.7%)	2 (7.1%)	2 (7.1%)	3 (10.7%)	2 (7.1%)	5 (17.9%)
**Study designs**								
Experimental study	1 (3.6%)	1 (3.6%)	2 (7.1%)	1 (3.6%)	2 (7.1%)	0 (0.0%)	1 (3.6%)	5 (17.9%)
Mixed-method study	4 (14.3%)	1 (3.6%)	3 (10.7%)	2 (7.1%)	1 (3.6%)	3 (10.7%)	3 (10.7%)	5 (17.9%)
Observational study	1 (3.6%)	0 (0.0%)	1 (3.6%)	1 (3.6%)	0 (0.0%)	0 (0.0%)	0 (0.0%)	1 (3.6%)
Pilot/feasibility study	1 (3.6%)	2 (7.1%)	3 (10.7%)	1 (3.6%)	3 (10.7%)	1 (3.6%)	2 (7.1%)	4 (14.3%)
Qualitative study	8 (28.6%)	4 (14.3%)	5 (17.9%)	4 (14.3%)	3 (10.7%)	6 (21.4%)	3 (10.7%)	10 (35.7%)
Secondary analysis	0 (0.0%)	1 (3.6%)	1 (3.6%)	1 (3.6%)	1 (3.6%)	0 (0.0%)	1 (3.6%)	3 (10.7%)

Note. Values are reported as contributing studies, n/28 (%). Percentages represent the proportion of the 28 included studies that contributed at least one barrier instance to the corresponding barrier domain within each clinical population or study design category. The final column reports the total number of unique studies reporting barriers within each clinical population or study design category. Because a single study could contribute to more than one barrier domain, domain-specific study counts should not be summed across rows or columns
